# Transcriptome and Metabolome Analyses Reveal Differences in Terpenoid and Flavonoid Biosynthesis in *Cryptomeria fortunei* Needles Across Different Seasons

**DOI:** 10.3389/fpls.2022.862746

**Published:** 2022-07-22

**Authors:** Yingting Zhang, Liwei Yang, Junjie Yang, Hailiang Hu, Guangqian Wei, Jiebing Cui, Jin Xu

**Affiliations:** Key Laboratory of Forest Genetics and Biotechnology of Ministry of Education, Co-Innovation Center for Sustainable Forestry in Southern China, College of Forestry, Nanjing Forestry University, Nanjing, China

**Keywords:** GC-MS, LC-MS, transcriptome, photosynthesis, flavonoid biosynthesis, terpenoid biosynthesis

## Abstract

*Cryptomeria fortunei* (Chinese cedar) has outstanding medicinal value due to its abundant flavonoid and terpenoid contents. The metabolite contents of *C. fortunei* needles differ across different seasons. However, the biosynthetic mechanism of these differentially synthesized metabolites (DSMs) is poorly understood. To improve our understanding of this process, we performed integrated non-targeted metabolomic liquid chromatography and gas chromatography mass spectrometry (LC-MS and GC-MS), and transcriptomic analyses of summer and winter needles. In winter, the *C. fortunei* needle ultrastructure was damaged, and the chlorophyll content and *F*_v_/*F*_m_ were significantly (*p* < 0.05) reduced. Based on GC-MS and LC-MS, we obtained 106 and 413 DSMs, respectively; based on transcriptome analysis, we obtained a total of 41.17 Gb of clean data and assembled 33,063 unigenes, including 14,057 differentially expressed unigenes (DEGs). Gene Ontology (GO) and Kyoto Encyclopedia of Genes and Genomes (KEGG) analyses showed that these DSMs*/*DEGs were significantly (*p* < 0.05) enriched in many biosynthesis pathways, such as terpenoids, photosynthates, and flavonoids. Integrated transcriptomic and metabonomic analyses showed that seasonal changes have the greatest impact on photosynthesis pathways, followed by terpenoid and flavonoid biosynthesis pathways. In summer Chinese cedar (SCC) needles, *DXS, DXR*, and *ispH* in the 2-methyl-pentaerythritol 4-phosphate (MEP) pathway and *GGPS* were highly expressed and promoted the accumulation of terpenoids, especially diterpenoids. In winter Chinese cedar (WCC) needles, 9 genes (*HCT, CHS, CHI, F3H, F3'H, F3'5'H, FLS, DFR*, and *LAR*) involved in flavonoid biosynthesis were highly expressed and promoted flavonoid accumulation. This study broadens our understanding of the metabolic and transcriptomic changes in *C. fortunei* needles caused by seasonal changes and provides a reference regarding the adaptive mechanisms of *C. fortunei* and the extraction of its metabolites.

## Introduction

Chinese cedar (*Cryptomeria fortunei*), a species endemic to China, has become one of the main ornamental and timber species in high-altitude areas in southern China due to its beautiful tree shape, rapid growth, excellent wood characteristics, and good application prospects. In addition, it serves as traditional medicinal material. Flavonoids and terpenoids are the main pharmacological components and show excellent detoxification, antioxidation, and antifungal and insecticidal properties (Cheng et al., 2005, 2009; Kofujita et al., 2006; Wang et al., 2006; Yoon et al., 2009; Xie et al., 2011, 2013); therefore, *C. fortunei* has been employed as a raw material in plant protection, cosmetic and pharmaceutical industries.

Seasonal changes (SCs) are the main components of global environmental changes because day length, light intensity, and temperature change dramatically during the annual cycle. Since most organisms have evolved in environments with natural seasons, the molecular systems underlying their environmental responses have adapted to SCs (Kudoh, 2016). In plants, SCs control life history, as manifested in many visible phenological events, such as germination, leaf expansion, flowering, and leaf shedding (Hepworth et al., 2015; Penfield and Macgregor, 2016). SCs cause corresponding physiological and cellular structural alterations in plants, such as changes in photosynthetic activity, soluble sugar and starch contents, and leaf ultrastructure (Tomkins et al., 1989; Keskitalo et al., 2005). SCs also cause changes in metabolites and other phytochemicals in trees and alter plant terpenoid production, alkene contents, and antioxidant and antibacterial properties (Kim et al., 2015; Budzinski et al., 2016; Hao et al., 2020). Terpene content is affected by SCs (Merk et al., 1988). For example, in Japanese cedar (*Cryptomeria japonica*) (Bang et al., 2020), the maximum content of terpenes was observed in summer. Among most other conifers, such as white spruce (*Pinus glauca*) (von Rudloff, 1972), blue spruce (*Pinus pungens*) (von Rudloff, 1975), and *Picea abies* (Schnwitz et al., 1990; Kamaityt-Bukelskien et al., 2021), there is a similar seasonal pattern of variation in the number of terpenes. Similarly, flavonoids, such as anthocyanins, vary greatly across different seasons (Zhang et al., 2022). For example, flavonoids are abundantly accumulated in *C. fortunei* needles under naturally low temperatures in winter (Zhang et al., 2022), while lodgepole pine (*Pinus contorta*) and jack pine (*Pinus bankiana*) exhibit a characteristic “purple color” in late autumn (Nozzolillo et al., 1990; Carnm et al., 1993). This color change is due to the accumulation of anthocyanin and cyanidin 3-glucoside in epidermal cells on the needle surface exposed to light (Carnm et al., 1993; Huner et al., 1998). A large number of anthocyanins accumulates in autumn and winter, which is beneficial for the resistance to low temperature and high visible light irradiance, which helps these trees smoothly survive the winter (Taulavuori et al., 2004). In addition, Xie et al. (2013) found that SCs affect the content of essential oils in *C. fortunei*. These results suggest that secondary metabolites in conifers vary across seasons (Gouvea et al., 2012); however, the underlying molecular regulatory mechanisms of SCs in the bioactive constituents (flavonoids and terpenoids) of conifer trees, especially *C. fortunei*, remain poorly understood.

Metabolomics specifically refers to the quantitative and qualitative analysis of a large number of low-molecular-weight (relative molecular weight <1,000) metabolites that exist in tissues, cells, or organisms to maintain their normal growth and function. Metabolite profiles directly reflect the overall physiological and biochemical status, which is conducive to studying the metabolic processes of plants and revealing the adaptability of plants to the environment (Nicholson et al., 1999; Sardans et al., 2011; van Dam and van der Meijden, 2011). In recent years, with the rapid development of liquid chromatography-mass spectrometry (LC-MS), gas chromatography–mass spectrometry (GC-MS), nuclear magnetic resonance (NMR), and other detection technologies, metabolomics technology has become a powerful analytical tool in the field of botany that is used for analyses of abiotic stress responses (Vaclavik et al., 2013; Xie et al., 2019), diversity (Fernandez et al., 2019), evolution (Deng et al., 2020), and growth and development regulation (Kazmi et al., 2017). GC-MS is generally suitable for the analysis of primary metabolites necessary for the survival and reproduction during plant growth and development, such as carbohydrates, proteins, lipids, and nucleic acids, while LC-MS is more suitable for evaluating a large group of plant secondary metabolites including alkaloids, flavonoids, and phenolic compounds (t'Kindt et al., 2009). Most studies use GC-MS or LC-MS technology to determine metabolites based on the characteristics of substances (Muráriková et al., 2017; Rehman et al., 2021); however, it is difficult to obtain all the metabolite information using a single detection technique due to the limitations and biases of the technique (Naz et al., 2014; Gonzalez-Dominguez et al., 2017). A few studies have performed multimetabolome analyses so far (Alves Filhoa et al., 2019; Zhou et al., 2020). For example, Lemos et al. (2015) used GC-MS to determine the essential oil composition of rosemary (*Rosmarinus officinalis*) and evaluated the extract composition by LC-MS and they found that the essential oils and extracts collected in summer had high concentrations of camphor and carnosic acid, thus showing higher antioxidant and antibacterial activities. This result provides the possibility of obtaining a comprehensive understanding of plant metabolites. The transcriptome is a collection of all transcripts produced by a species or a specific cell type, and it can be used to study gene function and structure at the overall level and reveal the molecular mechanisms of specific biological processes. A large number of functional gene annotation studies have now been performed in many plants based on transcriptome sequencing, as reported in Korean red ginseng (*Panax ginseng*) roots (Jayakodi et al., 2015) and living poplar (*Populus wutunensis*) (Zou and Jin, 2020). Due to the rapid progress in high-throughput data generation, multiomics integration methods are now being applied. Integrated metabolomics and transcriptomics methods are used for coexpression analyses of the causal relationships between differentially expressed unigenes (DEGs) and differentially synthesized metabolites (DSMs); combined with biological function analysis, such approaches can elucidate key genes and metabolites to further clarify the adaptation and regulation mechanisms of plants under specific physiological and ecological conditions. These hybrid platforms have made outstanding contributions to the discovery and identification of genes involved in processes, such as the biosynthesis of secondary metabolites in young and mature leaves of ginkgo (*Ginkgo biloba*) (Guo et al., 2020), flavonoid biosynthesis in the developing seeds of Tartary buckwheat (*Fagopyrum tataricum*) (Li et al., 2019), and carotenoid biosynthesis-related to the petal coloration of tulip tree (*Liriodendron tulipifera*) (Hao et al., 2020). Therefore, it is feasible to analyze the biosynthetic pathways of *C. fortunei* under different seasonal environmental conditions using integrated transcriptomics and metabolomics.

In this study, we used terpene-rich summer *C. fortunei* needles and flavonoid-rich winter *C. fortunei* needles as materials and comprehensively analyzed DEGs/DSMs and the corresponding enriched metabolic pathways through transcriptome and metabolomic analysis (LC- and GC-MS). On the basis of integrated omics analysis, we focused on terpenoid, photosynthetic, and flavonoid metabolism to reveal the molecular regulation of metabolic reactions in *C. fortunei* needles between seasons. To the best of our knowledge, this is the first molecular study revealing the response of the metabolites of *C. fortunei* to SCs, providing a reference for the selection and extraction of bioactive substances from *C. fortunei* and more useful information for the application of *C. fortunei* in the plant protection and in cosmetic and pharmaceutical industries.

## Materials and Methods

### Plant Materials and Growth Conditions

Three mature and healthy *C. fortunei* plants with an average age of 6–7 years from the Garden Experimental Teaching Center of Nanjing Forestry University (32°04′41″N, 118°48′43″E) were selected as the research materials. At 2 p.m. on 28 December 2019 (monthly average high/low temperature 11.65/3.13°C; actual sampling temperature 8°C) and 1 July 2021 (monthly average high/low temperature 31.16/25.55°C; actual sampling temperature 29°C), both of which were sunny days, needles were collected from secondary branches in the middle of the canopy as winter (WCC) and summer Chinese cedar (SCC) samples, respectively. A portion of the plant material was used for the observation of needle ultrastructure by transmission electron microscopy (TEM). Another portion of the material was immediately placed in an ice box and transferred to the laboratory (10 min) for pigment content determination. The remaining material was immediately placed in a 50 ml centrifuge tube, flash-frozen in liquid nitrogen, and transferred to a −80°C freezer for storage until metabolome and transcriptome analyses were performed.

### TEM Observations and Determination of Physiological Indicators

Needle material was immediately cut into 3 mm pieces and soaked in a vial containing penicillin in 2.5% (*v*/*v*) glutaraldehyde, followed by vacuum treatment and storage in a 4°C freezer. Material fixed with glutaraldehyde was washed twice (for 10 min each time) with 0.1 M phosphate buffer solution (PBS), fixed with osmium acid for 2 h, and washed again with 0.1 M PBS. Then, the material was dehydrated step by step with increasing concentrations of alcohol up to 100%, and different mixtures of acetone and epoxy resin (Poly/Bed 812; Polysciences, Warrington, PA, UK) were used for gradient infiltration (3:1, 1:1, 1:3, and 0:1, *v*/*v*). The samples were placed in a dry place overnight, and the needle ultrastructure was then observed with a JEM-1400 TEM system (JEOL Ltd., Tokyo, Japan) after embedding, slicing, and staining.

A fluorimeter (Dual-PAM/F-100, Walz, Effeltrich, Baden-Wuertenberg, Germany) was used to measure chlorophyll (chl) fluorescence parameters. Before the measurements, the third branches from the top of the secondary side branches were adapted to darkness for 30 min, after which saturated light (3,500 μmol m^−2^ s^−1^), actinic light (300 μmol m^−2^ s^−1^), and the dual channel mode (fluo + P700) were applied to measure the maximum quantum yields of Photosystem II (PS_II_ and *F*_v_/*F*_m_).

The contents of chl and carotenoids were determined and calculated using the Lichtenthaler and Wellburn (1983) method. Specifically, 0.1 g of needles, 2 ml of 96% ethanol, and small amounts of quartz sand and calcium carbonate powder were added to a precooled mortar, ground into a homogenous slurry, and transferred to a centrifuge tube for centrifugation at the 5,000 rpm for 20 min until the tissue turned white. The supernatant was diluted to 10 ml and measured at wavelengths of 663, 645, and 470 nm.

### GC-MS Analysis

An accurately weighed 80 mg needle sample, 20 μl of an internal standard (0.06 mg mL^−1^ L-2-chlorophenylalanine, methanol configuration), 2 small steel balls, and 360 μl of precooled methanol were added to a 1.5 ml centrifuge tube, ground at 60 Hz for 2 min and ultrasonically extracted in an ice-water bath for 30 min. After adding 200 μl of chloroform and 400 μl of water, the sample was vortexed in a vortexer for 2 min, ultrasonically extracted in an ice-water bath for 30 min, left to stand at −20°C for 30 min, and centrifuged at 4°C at 13,000 rpm for 10 min. The supernatant (150 μl) was transferred to a glass derivatization vial and evaporated with a centrifugal concentration dryer. Following the addition of 80 μl of a 15 mg mL^−1^ methoxyamine hydrochloride pyridine solution to the glass vial, the sample was vortexed for 2 min and incubated at 37°C for 90 min with shaking. Then, 50 μl of N,O-bis(trimethylsilyl)trifluoroacetamide (BSTFA) [containing 1% of trimethylchlorosilane (TMCS)] and 20 μl of n-hexane were added, and the sample was vortexed for 2 min, reacted at 70°C for 60 min, and placed at 25°C for 30 min for GC-MS metabolomic analysis.

The derived samples were analyzed by GC-MS (Agilent Technologies Inc., CA, USA) coupled with 5977A and 7890B systems. A DB-5MS capillary column (30 m × 0.25 mm × 0.25 μm, Agilent J&W Scientific, Folsom, CA, USA) was used to separate derivatives. Helium (>99.999%) was used as the carrier gas and was passed through the column at a constant flow rate of 1 ml min^−1^; the injector temperature was maintained at 260°C, and the volume was 1 μl in splitless mode. The temperature program mode was as follows: the temperature of the column thermostat was held at 60°C for 0.5 min, increased to 210°C at a rate of 8°C min^−1^, increased to 270°C at 15°C min^−1^, increased to 305°C at 20°C min^−1^, and held at that temperature for 5 min. The MS electron impact ion source and quadrupole temperatures were set to 230 and 150°C, respectively, and the electron energy was 70 eV. The mass data were collected in full scan mode (m/z 50–500), and the solvent delay time was set to 5 min.

The raw data were transformed *via* the software Analysis Base File Converter and imported into MS-DIAL software for peak detection, peak identification, MS2Dec deconvolution, characterization, peak alignment, wave filtering, and missing value interpolation. Metabolite characterization was based on the LUG database, and a data matrix was derived. In each sample, all peak signal intensities were segmented and normalized based on the internal standards with a relative standard deviation (RSD) > 0.3 after screening. The data matrix was obtained after further redundancy removal and peak merging.

### LC-MS Metabolomic Analysis

An accurately weighed 80 mg sample of needles, 20 μl of an internal standard, 800 μl of methanol-water (*v*:*v* = 7:3), and 2 precooled small steel balls were added to a 1.5 ml centrifuge tube, ground at 60 Hz for 2 min, ultrasonically extracted in an ice-water bath for 30 min, left to stand overnight at −20°C, and centrifuged at 4°C at 13,000 rpm for 10 min. The supernatant (150 μl) was aspirated with a syringe, filtered through a 0.22 μm organic-phase pinhole, transferred to an LC injection vial, and stored at −80°C until LC-MS analysis.

An ACQUITY UPLC I-Class system (Waters Co., Milford, USA) and VION IMS QTOF mass spectrometer (Thermo Fisher Scientific) with a 45°C ACQUITY UPLC HSS T3 (100 × 2.1 mm, 1.8 μm) column were used to analyze metabolic profiles in ESI positive and negative ion modes, respectively. Water and acetonitrile (both containing 0.1% of formic acid) were used as mobile phases A and B, respectively, and the elution program was as follows: 0.01 min, 95% A; 4 min, 70% A; 8 min, 50% A; 10 min, 20% A; 14 min, 0% A; and 15 min, 95% A. The injection volume was 2 μl, and the flow rate was 0.35 ml min^−1^. Data acquisition was performed in mass scan range (m/z 100 to 1200) mode, and the mass spectrometry parameters were as follows: resolution (full scan), 70,000; resolution (HCD MS/MS scans), 17,500; spray voltage, 3800 V (+) and 3200 V (-); sheath and aux gas flow rate, 40 and 10 Arb; and capillary temperature, 320°C.

The raw LC-MS data were preprocessed by Progenesis QI v2.3 (Nonlinear, Dynamics, Newcastle, UK) with the following parameters: precursor and product tolerance, 5 and 10 ppm; and production threshold, 5%. Compounds were qualitatively identified based on accurate masses, secondary fragments, isotope distributions, and the protein model database (PMDB). More than 50% of the extracted data were further processed by deleting peaks with missing values (value = 0) and replacing them with half of the minimum value, and compounds with scores <36 (full score of 60) were considered inaccurate qualitative results and deleted.

The GC-/LC-MS data matrix was imported into R to perform principal component analysis (PCA) to observe the distribution stability between samples throughout the analysis process and to perform (orthogonal) partial least squares discriminant analysis ((O)PLS-DA) to distinguish the overall differences in the metabolic profiles between groups. To prevent overfitting, 7-fold cross-validation and 200 response permutation tests (RPTs) were used to evaluate the quality of the model. The values of variable importance of projection (VIP) were obtained based on the OPLS-DA model, where metabolites with a VIP > 1 were considered potential DSMs and were further verified by *t*-tests to determine whether the differences in metabolites between groups were significant (*p* < 0.05).

### Transcriptomic Analysis

Total RNA was extracted using the mirVana™ miRNA ISOlation Kit (Ambion Life Technologies, Austin, TX, USA) according to the manufacturer's instructions. Library construction and sequencing were completed by OE Biotech Co., Ltd. (Shanghai, China).

Six RNA libraries (SCC-1, SCC-2, SCC-3, WCC-1, WCC-2, and WCC-3) were constructed and analyzed. A total of 43.02 Gb of raw bases were obtained (Supplementary Table 1), and these raw base data were submitted to the NCBI Sequence database [PRJNA793065 (SUB10872191) and PRJNA697258 (SUB9488615 (SAMN17672905–07))]. The raw data generated *via* high-throughput sequencing were subjected to further quality filtering. That is, high-quality clean reads were obtained using Trimmomatic (Bolger et al., 2014) for quality control to remove connectors and filter out low-quality and N bases. These clean reads were *de novo* assembled into unigene sequences using Trinity v2.4 (Grabherr et al., 2013) based on the paired-end splicing method, and the longest transcript was defined as a unigene according to sequence similarity and length. The functions of the unigenes were annotated by the alignment of the unigenes with the non-redundant (Nr), eukaryotic orthologous groups (KOG) of proteins, Gene Ontology (GO), SwissProt, eggNOG, and Kyoto Encyclopedia of Genes and Genomes (KEGG) databases using Diamond (Buchfink et al., 2015) and the Pfam database using HMMER (Mistry et al., 2013). Fragments per kilobase of exon model per million mapped fragments (FPKM) values (Trapnell et al., 2010) were obtained by calculating the number of unigene reads in each sample using eXpress (Roberts and Pachter, 2013), and the data were further standardized using the estimateSizeFactors function of the DESeq R package (Anders and Huber, 2016). The *p-* values and fold changes were calculated using nbinomTest, and DEGs were identified according to the thresholds of a *p* < 0.05 and |log2FoldChange| > 1. GO and KEGG (Kanehisa et al., 2008) enrichment analyses of DEGs were performed to determine the main affected biological functions or pathways. Additionally, hierarchical clustering analysis was performed to reveal the expression patterns of DEGs among different samples (https://cloud.oebiotech.cn/task/).

### Combined Analysis of DSMs and DEGs

Based on all of the DEGs and DSMs annotated with KEGG pathways, Pearson's correlation test was performed to identify the correlations between the metabolites and associated genes (*p* < 0.05) (https://cloud.oebiotech.cn/task/). In addition, DEGs and DSMs were mapped to the KEGG pathway database to obtain their common pathway information.

### Quantitative Real-Time Polymerase Chain Reaction

A total of 1 μg of each qualified total RNA sample was reverse transcribed with the HiScript III RT SuperMix Kit (Vazyme Biotech Co., Ltd., Nanjing, Jiangsu, China) according to the manufacturer's protocol, and these complementary DNA (cDNA) templates were stored in a freezer at −80°C. Eighteen candidate DEGs in the photosynthesis, flavonoid, and terpenoid pathways were randomly selected, and the corresponding gene-specific primers (Supplementary Table 2) were designed, and all primers were synthesized by Tsingke Biotech Co., Ltd. (Nanjing, Jiangsu Province, China). The expression of candidate genes was detected by qRT-PCR using the ChamQTM SYBR® qPCR Master Mix Kit (Low ROX Premixed) (Vazyme Biotechnology Co.), and the reaction system (20 μl) was as follows: 10 μl of 2 × ChamQ SYBR qPCR Master Mix (Low ROX Premixed); 2 μl of 10-fold-diluted cDNA (cDNA:water, 1:9, *v*:*v*); 0.8 μl of each specific primer pair (10 μM forward and reverse primers); and 7.2 μl of ddH_2_O. The qRT-PCR was performed on an Applied Biosystems (ABI) 7500 fast real-time PCR system (ABI, Foster City, CA, USA), and the amplification procedure was as follows: 95°C for 30 s; 40 cycles of denaturation at 95°C for 10 s and annealing and extension at 60°C for 30 s, followed by melting curve analysis according to the default settings of the instrument. The 2^−ΔΔCt^ method (Livak and Schmittgen, 2001) was used to calculate gene expression levels with the *Actin, cyclophilin* (*CYP*), and *ubiquitin-conjugating enzyme* (*UBC*) genes as references (Zhang et al., 2021). All reactions in all experiments were repeated three times.

## Results

### Needle Ultrastructure and Physiological Indices Show Significant Changes

**Figure 1:**
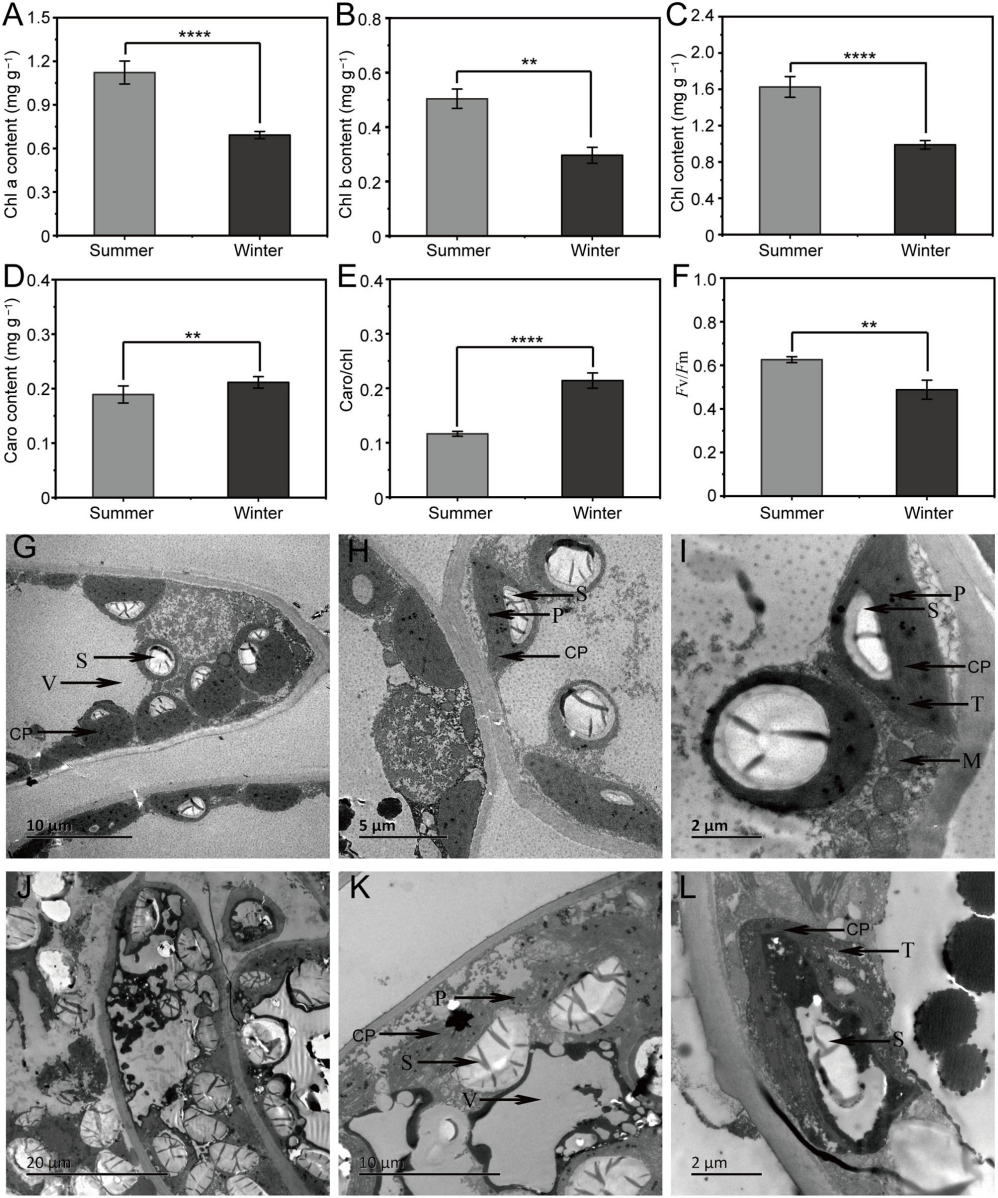
Physiological indices and needle ultrastructure associated with seasonal changes in *Cryptomeria fortunei* needles. **(A)** Chlorophyll (chl) a content; **(B)** chl b content; **(C)** chl contents; **(D)** carotenoid content; **(E)** carotenoid/chl; and **(F)** *F*_v_/*F*_m_. The average ± standard deviation is displayed, *n* = 6. Student's *t-*test was adopted to define the statistical significance of the difference, **, *p* < 0.01 and ****, *p* < 0.0001. Representative image of the needle ultrastructure of summer **(G–I)** and winter **(J–L)** *C. fortunei* needles. Bars = 20, 10, 5 or 2 μm. CP, chloroplast; CW, cell wall; V, vacuole; S, starch granule; M, mitochondria; T, thylakoids; and P, plastoglobuli.

Compared with the SCC results, the chl levels (chl a, b, and a + b) and *F*_v_/*F*_m_ in WCC samples were significantly (*p* < 0.05) reduced, while the carotenoid levels and carotenoid/chl levels were significantly (*p* < 0.05) increased (Figures 1A–F). The observation of needle ultrastructure showed obvious differences in the structure of needle chloroplasts across different seasons. The SCC needle chloroplast structure was relatively stable, surrounding the inner surface of the cell, and chloroplast basal lamellae were densely stacked; however, the WCC needle chloroplast structure was slightly deformed, the thylakoid structure was loose, and the number of chloroplast basal grains and the number of stacked layers was low (Figures 1G–L). These results indicate that the local destruction of the WCC chloroplast structure reduces the pigment content, resulting in different morphological phenotypes of *C. fortunei*.

### GC-MS-Based Metabolomics Reveals Metabolite Changes in *C. fortunei* Needles Across Different Seasons

**Figure 2:**
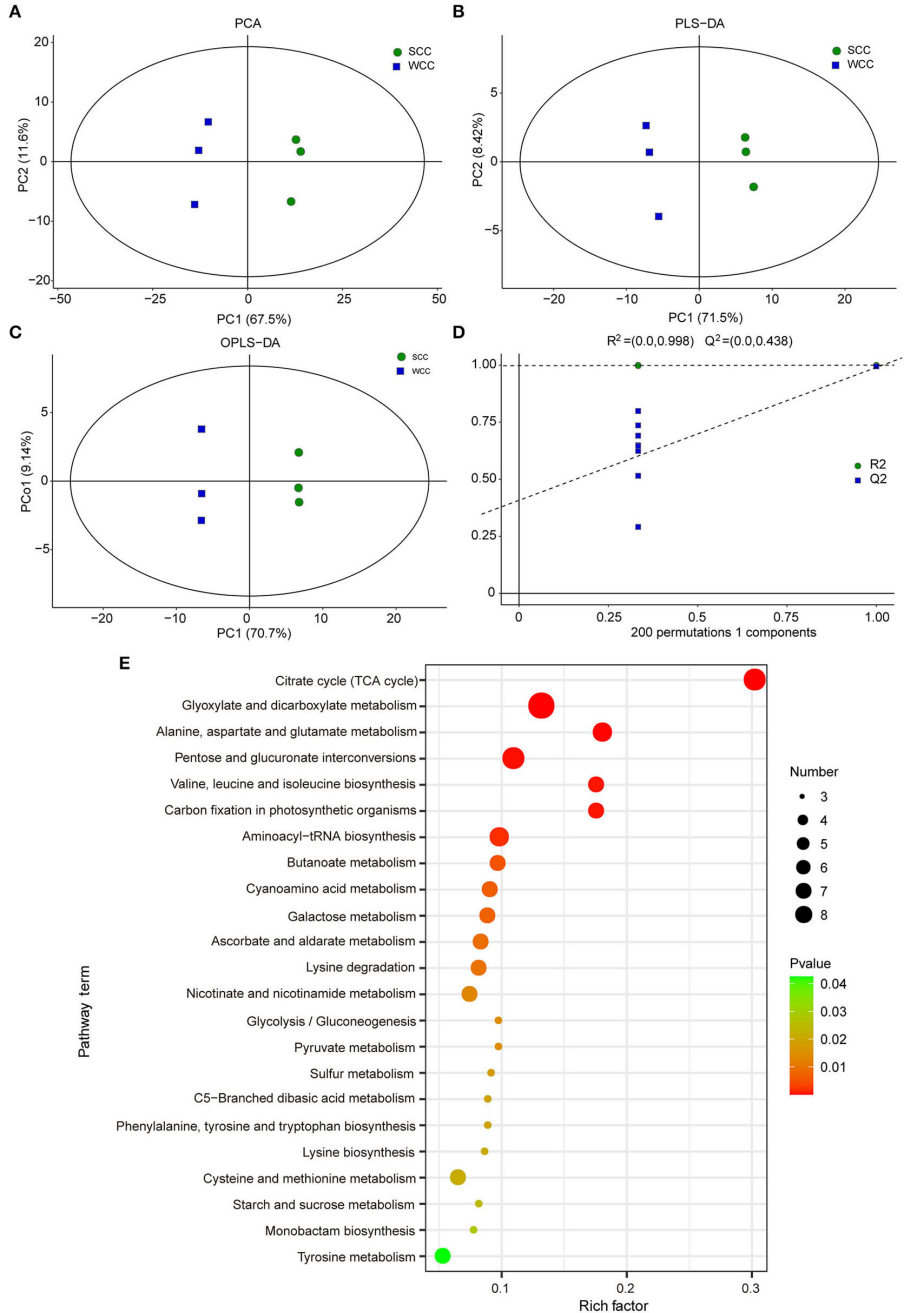
Multivariate statistical analysis of the differentially synthesized metabolites (DSMs) based on gas chromatography–mass spectrometry (GC-MS). **(A)** Plots of principal component analysis (PCA); **(B)** partial least squares discriminant analysis (PLS-DA); and **(C)** orthogonal PLS-DA (OPLS-DA). The *x*- and *y*-axes represent the first (PC1) and second (PC2)/orthogonal (PCo1) principal components, respectively; SCC and WCC represent summer and winter *C. fortunei* needles, respectively. **(D)** The 200-response sorting tests of the OPLS-DA model. **(E)** KEGG analysis of DSMs. The *x*- and *y*-axes represent the enrichment factor and pathway term, respectively. The colors and sizes of the dots represent the significance (*p*) and number of metabolites, respectively.

We analyzed the non-targeted metabolome of *C. fortunei* needles using GC-MS to investigate the metabolic differences between seasons. Visual inspection of the total ion chromatograms (TICs) of all samples showed that the instrument exhibited strong analytical signals, a high peak capacity, and good retention time reproducibility (Supplementary Figure 1). A total of 277 metabolites were identified in *C. fortunei* needles; the most abundant metabolite group was “carbohydrates and carbohydrate conjugates,” accounting for 19.49% of all metabolites, followed by “amino acids, peptides, and analogs” (13.00%) (MTBLS4099) (Haug et al., 2019). Based on these metabolites, PCA and (O)PLS-DA accurately divided all samples into 2 clusters, reflecting the obvious differences between summer and winter needles (Figures 2A–C). A 200 RPT was further performed, and the *R*^2^ value was 0.998. The *Q*^2^ values of all blue points on the left were lower than those of the original points on the right, indicating that the data calculated by the OPLS-DA model were reliable (Figure 2D). A combination of single- and multidimensional analyses was used to screen DSMs between the SCC and WCC groups, and 106 DSMs (25 down- and 81 upregulated in WCC needles) were obtained (Supplementary Figure 2). Among these DSMs, the chlorogenic acid, epicatechin, and citric acid contents in WCC needles were 108.121-, 38.959- and 25.854-fold higher, respectively, than those in SCC needles (Supplementary Table 3). A total of 63 DSMs were enriched in 69 metabolic pathways (Supplementary Table 4), including 23 significant metabolic pathways (Figure 2E). Compared with the SCC results, the upregulated DSMs in WCC needles were significantly (*p* < 0.05) enriched in the “citrate cycle (TCA cycle),” “glyoxylate and dicarboxylate metabolism,” and “lysine degradation,” while the downregulated DSMs were significantly (*p* < 0.05) enriched in “aminoacyl-tRNA biosynthesis,” “valine, leucine, and isoleucine biosynthesis” and “cysteine and methionine metabolism” (Supplementary Figure 3).

### LC-MS-Based Metabolomics Reveals Metabolite Changes in *C. fortunei* Needles Across Different Seasons

**Figure 3:**
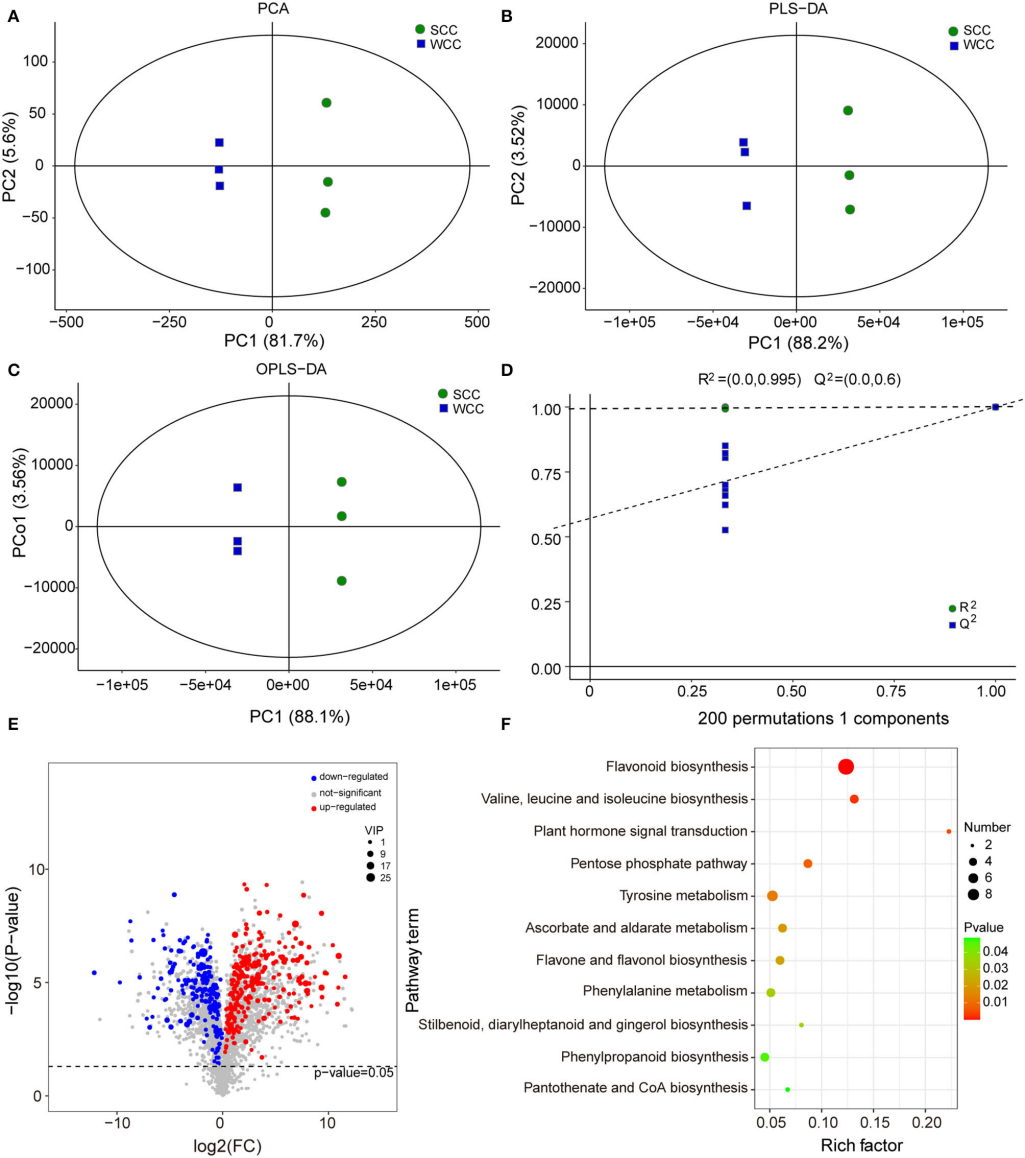
Multivariate statistical analysis of the differentially synthesized metabolites (DSMs) based on liquid chromatography-mass spectrometry (LC-MS). **(A)** Plots of principal component analysis (PCA); **(B)** partial least squares discriminant analysis (PLS-DA); and **(C)** orthogonal PLS-DA (OPLS-DA). The *x*- and *y*-axes represent the first (PC1) and second (PC2)/orthogonal (PCo1) principal components, respectively. SCC and WCC represent summer and winter *C. fortunei* needles, respectively. **(D)** The 200-response sorting tests of the OPLS-DA model. **(E)** Volcano plot of DSMs. Each point in the volcano plot represents a metabolite, the abscissa represents the logarithm of the quantitative difference multiplied by a metabolite in two samples, and the ordinate represents the significance level of DSMs. The blue/red dots in the figure represent down/upregulated DSMs, and the gray dots represent metabolites that were detected but not significant. **(F)** KEGG analysis of DSMs. The *x*- and *y*-axes represent the enrichment factor and pathway term, respectively. The colors and sizes of the dots represent the significance (*p*) and number of metabolites, respectively.

To further investigate the metabolic differences between seasons, we analyzed the non-targeted metabolome of *C. fortunei* needles using LC-MS. The base peak diagrams of all samples showed that the instrument exhibited strong analytical signals, a high peak capacity, and good retention time reproducibility (Supplementary Figure 4). We identified 24,318 metabolites, including 2,880 metabolites (991 neg and 1,989 pos) with definite formulas (MTBLS4099) (Haug et al., 2019). Five of these metabolites had more than 100 specific metabolites [i.e., “carbohydrates and carbohydrate conjugates” (265), “flavonoid glycosides” (214), “sesquiterpenoids” (167), “amino acids, peptides, and analogs” (126) and “triterpenoids” (121)]. For these metabolites, PCA and (O)PLS-DA analyses showed that significant differences existed between SCC and WCC needles (Figures 3A–C), and the accuracy of the OPLS-DA model was validated by 7-fold cross-validation and 200 RPT (Figure 3D). Using a combination of multi- and single-dimensional analyses, a total of 413 DSMs were obtained (Supplementary Figure 5; Figure 3E). Among these DSMs, 44 DSMs were enriched in 45 pathways (Supplementary Table 5), including 11 significant metabolic pathways (Figure 3F). In WCC needles, the upregulated DSMs were significantly (*p* < 0.05) enriched in “flavonoid biosynthesis” (FBP), “flavone and flavonol biosynthesis,” and “phenylpropanoid biosynthesis,” while the downregulated DSMs were significantly (*p* < 0.05) enriched in the “plant hormone signal transduction,” “valine, leucine, and isoleucine biosynthesis,” “tyrosine metabolism,” “pentose phosphate,” “cyanoamino acid metabolism,” and “terpenoid backbone biosynthesis” (TBBP) pathways (Supplementary Figure 6).

### Assembly and Annotation of the *C. fortunei* Transcriptome

After filtering out low-quality sequences, 41.17 Gb of clean data were obtained; the effective amount of data from each sample ranged from 6.35 to 7.44 Gb, the Q30 of each sample was above 95.36%, and the average GC content was 44.67% (Supplementary Table 1). By assembling these clean reads, we obtained 33,063 unigenes (Supplementary Tables 6, 7), with total, average, and N50 lengths of 42,468,815, 1,284.48, and 1,965 bp, respectively (Supplementary Table 8). The lengths of 6,822 (20.63%) of the single unigenes were >2 kb (Supplementary Figure 7).

A total of 21,901 unigenes were annotated in 7 public databases through BLAST searches. The greatest number of unigenes was annotated in the NR library (21,309), accounting for 64.45% of the total, followed by eggNOG (19,833, 59.99%), Pfam (16,691, 50.48%), and SwissProt (16,470, 49.81%) (Supplementary Table 9). Fifty-two GO terms associated with 13,654 annotated unigenes (41.30%) were divided into 3 categories [i.e., biological process (BP, 23), cellular component (CC, 13), and molecular function (MF, 16)] (Supplementary Figure 8). The largest subcategory of BP was “cellular process,” and “cell” and “binding” were the largest subcategories of CC and MF, respectively (Supplementary Figure 8). KEGG annotation was used to determine the synthetic pathways of bioactive components in *C. fortunei* by mapping the assembled unigenes, and 4,640 (14.03%) unigenes were annotated into 5 categories, including 20 subcategories. Among these pathways, “carbohydrate metabolism” (732), “translation” (699), “folding, sorting, and degradation” (509), “lipid metabolism,” (435) and “amino acid metabolism” (427) were associated with significantly greater numbers of single genes than the other pathways (Supplementary Figure 9). We further performed a PCA of all unigenes, compared the expression patterns of SCC and WCC, and found that the same groups of samples were classified together and showed a high degree of similarity (≥ 0.9249) (Supplementary Figure 10).

### GO and KEGG Enrichment Analysis of DEGs

**Figure 4:**
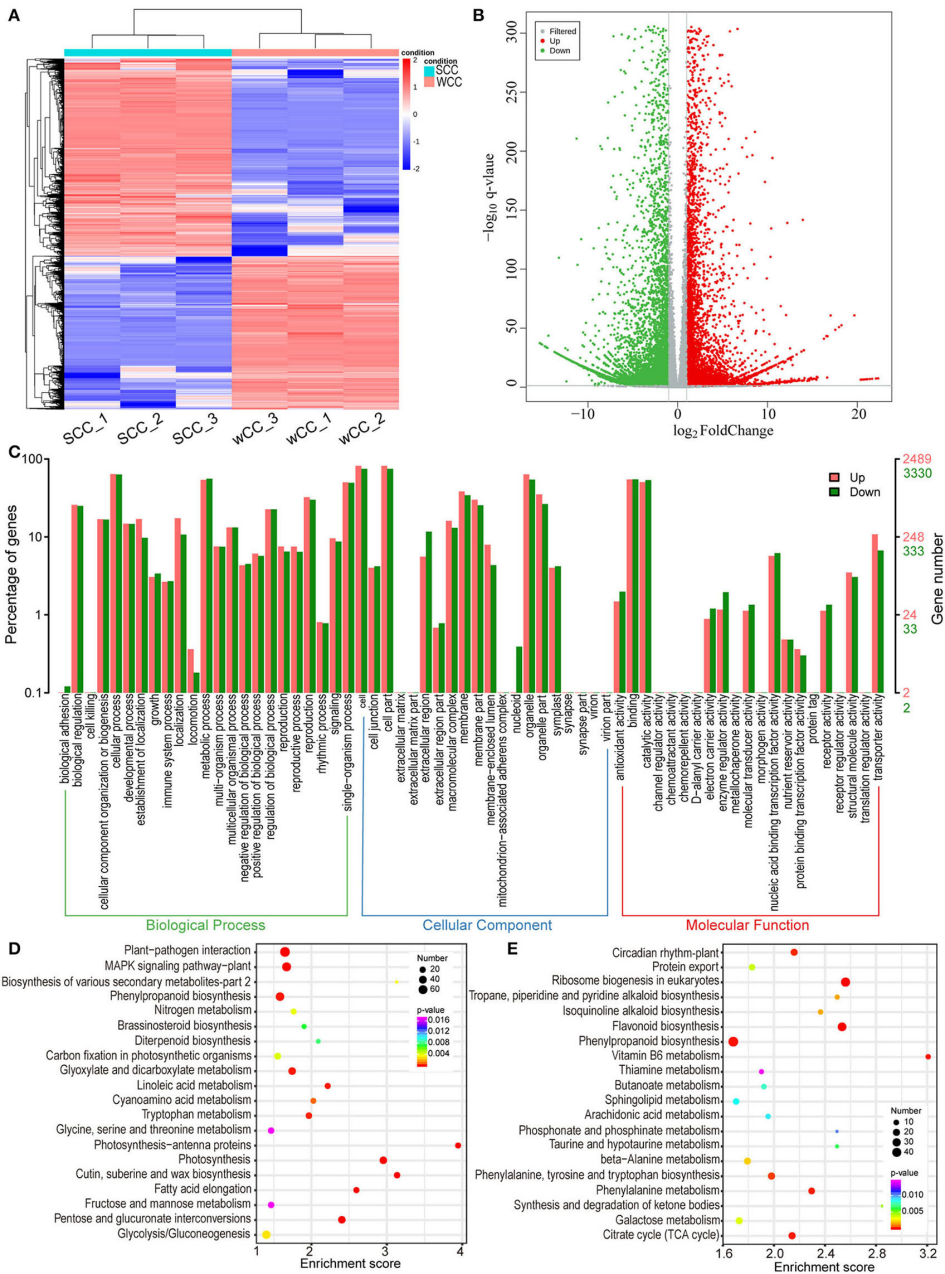
Functional classification of differentially expressed unigenes (DEGs). **(A)** Hierarchical clustering heatmap of DEGs. Each column and row represent a sample and a DEG, respectively, and the colors indicate the expression levels of DEGs. SCC and WCC, summer and winter Chinese cedar needle samples, respectively, and SCC_1, SCC_2, and SCC_3 represent 3 repetitions. **(B)** Volcano plot of DEGs. Each point in the volcano plot represents a unigene, the abscissa represents the logarithm of the quantitative difference multiples of a unigene in two samples, and the ordinate represents the significance level of DEGs. The blue/red dots in the figure represent down/upregulated DSMs, and the gray dots represent metabolites that were detected but not significant. **(C)** Comparison of the distribution of DEGs at the GO level. Red/green indicates GO entries enriched for up/downregulated DEGs; the *x*-axis is the name of the entry, and the *y*-axis represents the number of unigenes in the corresponding entry and their percentages. KEGG enrichment analysis of downregulated **(D)** and upregulated **(E)** DEGs in WCC needles. The *x*- and *y*-axes represent the enrichment score and pathway term, respectively. The colors and sizes of the dots represent the significance (*p*) and number of unigenes, respectively.

A total of 14,057 DEGs (6,137/7,920, up/downregulated in WCC needles) were identified between the WCC and SCC samples (Figures 4A,B). Among these DEGs, 5,819 were annotated with GO terms, among which the most enriched subcategory was “cell,” followed by “cell part”; the two most annotated subcategories in BP, CC, and MF were “cellular component organization or biogenesis” and “metabolic process”; “cell” and “cell part”; and “catalytic activity” and “binding,” respectively (Figure 4C).

To gain insight into the synthesis of specific metabolites, related gene functions, and multigene interactions, we further performed a KEGG enrichment analysis. The downregulated DEGs were annotated into 29 significant (*p* < 0.05) pathways. Among these pathways, “photosynthesis” (PP) and “photosynthesis-antenna proteins” (PPP) were the 2 most significant pathways; “carbon fixation in photosynthetic organisms,” “brassinosteroid biosynthesis,” and “diterpenoid biosynthesis” were also significantly enriched (Figure 4D). The upregulated DEGs were annotated to 18 significant (*p* < 0.05) pathways, among which “ribosome biogenesis in eukaryotes” and FBP were the two most significant pathways (Figure 4E).

### Integrated Analysis of the Transcriptome and Metabolome

**Figure 5:**
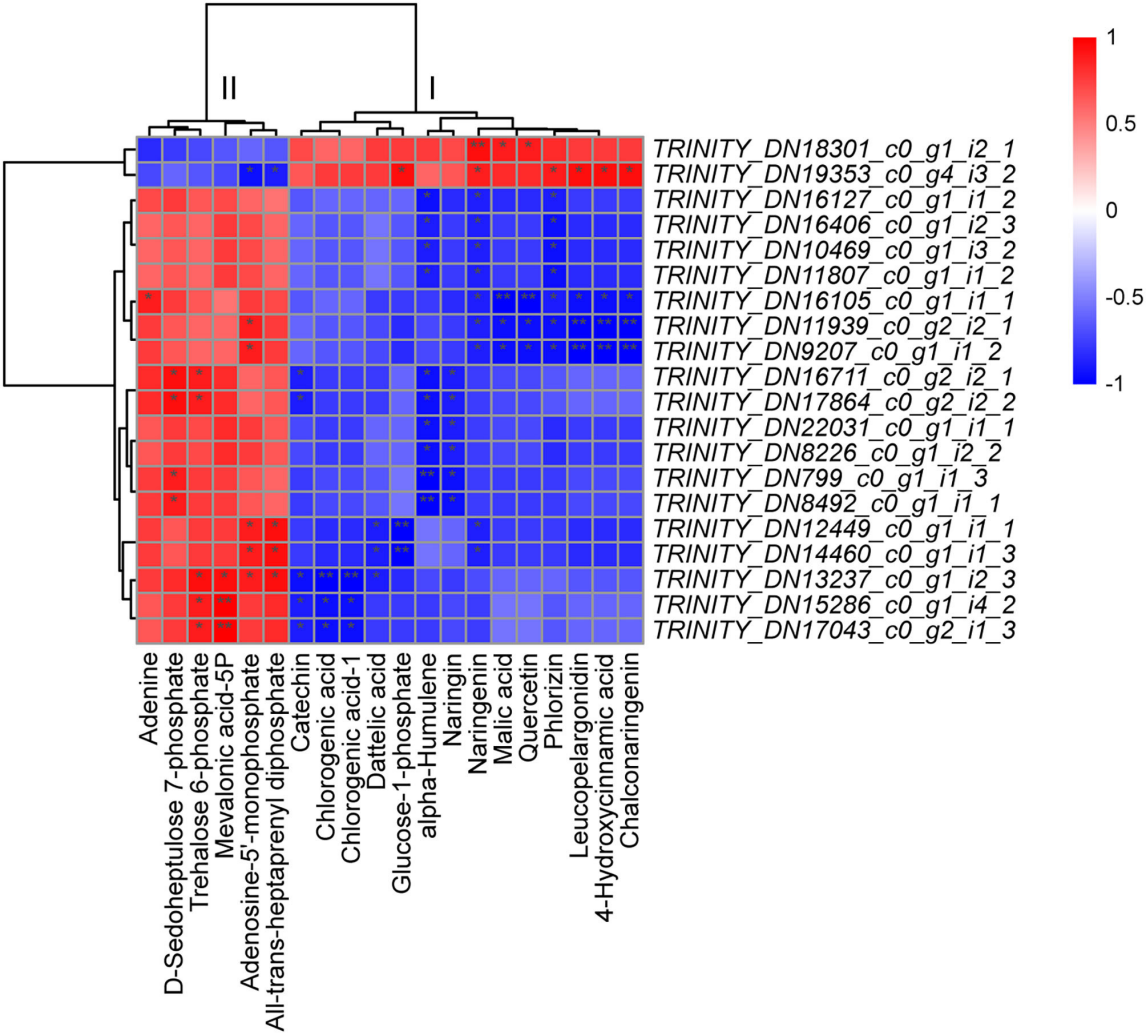
Correlation analysis of the top 20 differentially expressed unigenes (DEGs) and differentially synthesized metabolites (DSMs). Red/blue indicates a positive/negative correlation, and a deeper color indicates stronger correlations; among them, * represents a significant correlation (*p* ≥ 0.5 or *p* ≤ –0.5). ** represents a very significant correlation (*p* = 1.0 or 1.0).

To further explore the potential regulatory mechanisms, we performed Spearman's correlation analysis between the DEGs (2,044) and DSMs (107) annotated with KEGG pathways. Among the top 20 unigenes, 14 unigenes (70%) were related to PP/PPP, 3 unigenes were related to “arginine biosynthesis” and “carbon fixation in photosynthetic organisms,” and 1 unigene was related to “MAPK signaling pathway-plant/FBP/RNA transport” (Supplementary Table 10). Among the top 20 metabolites, 5, 4, 2, 6, and 1 were related to FBP, TBBP, “carbon fixation in photosynthetic organisms and starch and sucrose,” “amino acid,” and “TCA/biosynthesis of unsaturated fatty acids/ascorbate and aldarate metabolism,” respectively (Supplementary Table 10). The metabolites were clearly divided into two categories: I (including 14 types; i.e., catechin, 2 chlorogenic acids, dattelic acid, glucose-1-phosphate, alpha-humulene, naringin, naringenin, malic acid, quercetin, phlorizin, leucopelargonidin, 4-hydroxycinnamic acid, and chalconaringenin) and II (the remaining 6 types) (Figure 5). The same category of metabolites was positively correlated; in contrast, different categories of metabolites were generally negatively correlated (Figure 5). TRINITY_DN18301_c0_g1_i2_1 and TRINITY_DN19353_c0_g4_i3_2 were positively correlated with category I metabolites and negatively correlated with category II metabolites (Figure 5). We further speculate that the seasonal metabolic differences in *C. fortunei* are mainly related to PP, followed by TBBP and FBP.

### Identification of Unigenes Related to PP, TBBP, and FBP

**Figure 6:**
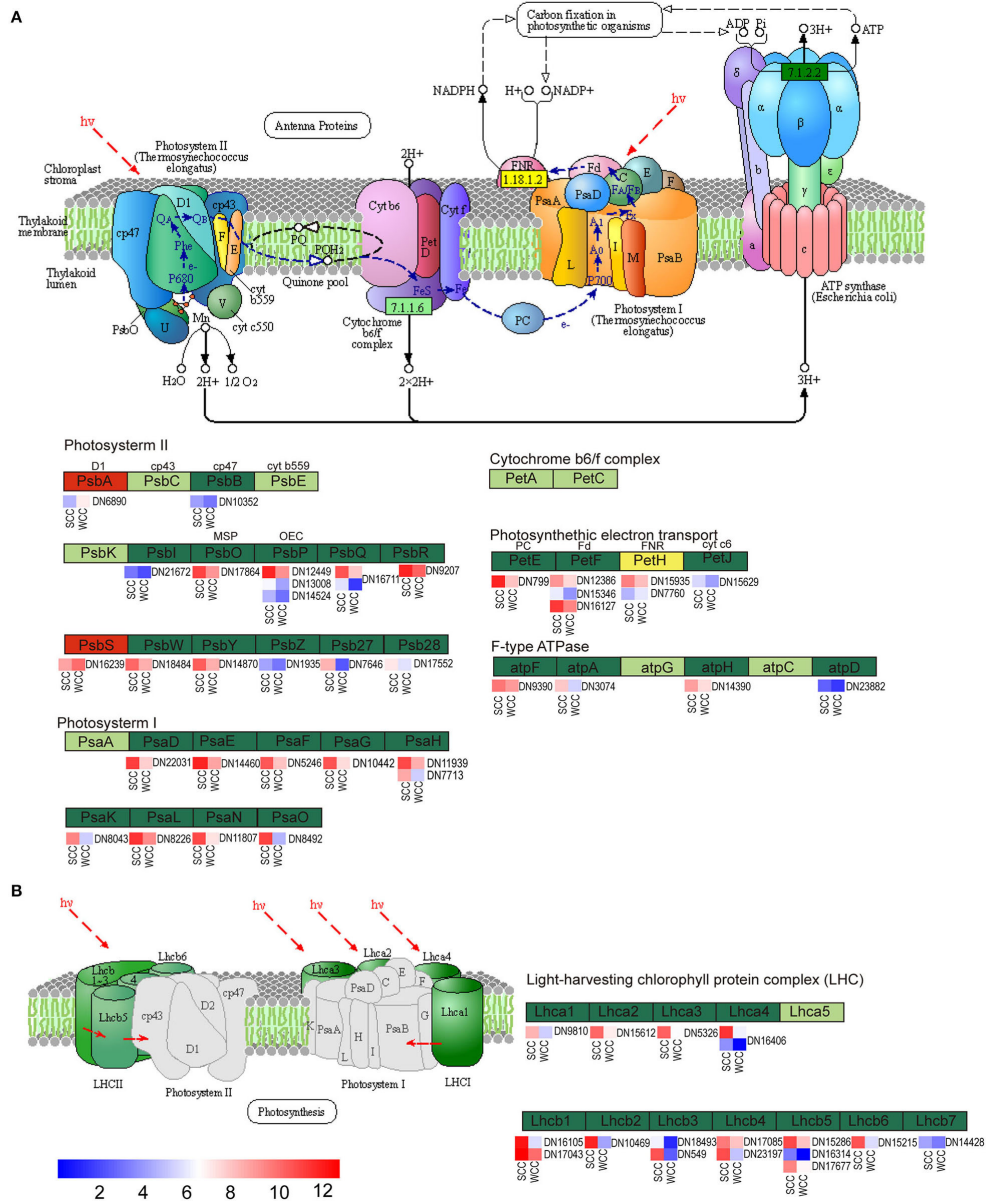
Metabolic pathway map of photosynthesis-related biosynthesis. **(A)** Photosynthesis pathway; **(B)** photosynthesis-antenna protein pathway. Dark red/green represents up/downregulated genes, yellow represents genes that were both up- and downregulated, and small red/blue squares indicate the expressed levels of unigenes. SCC and WCC, summer and winter Chinese cedar needle samples, respectively. The abbreviations of each gene are as follows: F-type ATPase (*atpF, A, H*, and *D*); light-harvesting chlorophyll protein complex (*Lhca1–4* and *Lhcb1–7*); photosynthetic electron transport (*PetE, F, H*, and *J*); photosystem I (*PsaD, E, F, G, H, K, L, N*, and *O*); and photosystem II (*PsbA, B, I, O, P, Q, R, W, Y, Z, 27*, and *28*).

According to our aforementioned results obtained using metabolomics combined with transcriptomics, the two samples of *C. fortunei* needles differed significantly in the expression of PP, TBBP, and FBP components (Supplementary Table 10). A total of 56 DEGs were related to PP (Figure 6). While PSII proteins, PsbA and PsbS and petH were upregulated in WCC needles, the remaining 53 DEGs, including genes related to PSII (psbB, I, O–R, W, Y, 27 and 28), PSI (psaD–H, K, L, N, and O), photosynthetic electron transport (petE, F, and J), F-type ATPase (atpA, F, D, and H), and light-harvesting chl (LHC) protein complexes (Lhca1–4 and Lhcb1–7), were significantly (*p* < 0.05) downregulated in WCC needles (Supplementary Table 11).

**Figure 7:**
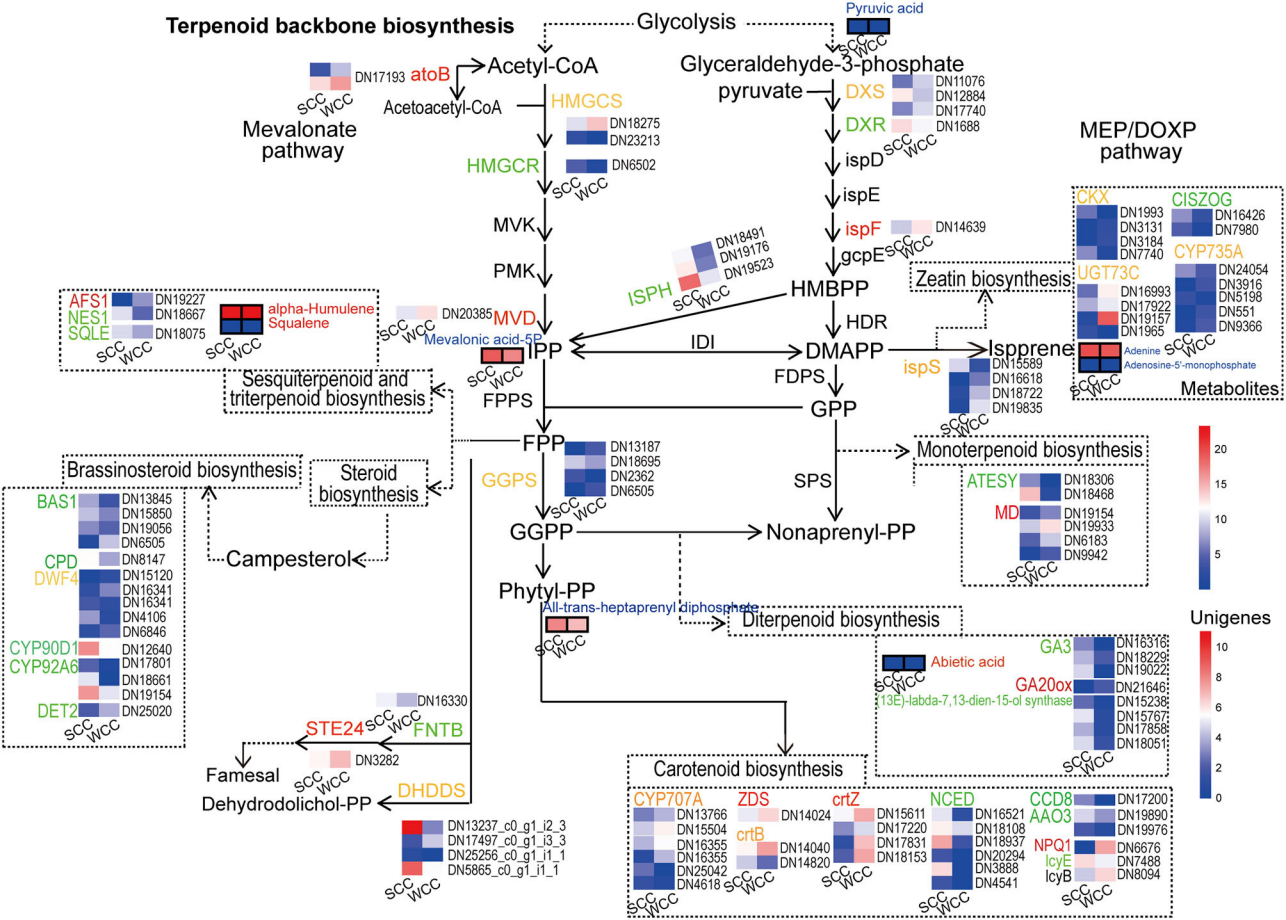
Metabolic pathway map of terpenoid-related biosynthesis pathways. SCC and WCC represent summer and winter *C. fortunei* needles, respectively. Small squares without/with lines indicate the expressed levels of unigenes/metabolites, and red/green/yellow text indicates up/down/both up- and downregulated genes or metabolites in WCC needles. The abbreviation of each gene is as follows: *AAO3, abscisic-aldehyde oxidase*; *AFS1, alpha-farnesene synthase*; *ATESY, alpha-terpineol synthase*; *atoB, acetyl-CoA acetyltransferase*; *BAS1, PHYB activation tagged suppressor 1*; *CCD8, carlactone synthase*; *CISZOG, cis-zeatin O-glucosyltransferase*; *CKX, cytokinin dehydrogenase*; *CPD, cytochrome P450 family 90 subfamily A polypeptide 1*; *crtB, 15-cis-phytoene synthase*; *crtZ, beta-carotene 3-hydroxylase*; *CYP707A, (+)-abscisic acid 8'-hydroxylase*; *CYP735A, cytokinin trans-hydroxylase*; *CYP90D, 1,3-epi-6-deoxocathasterone 23-monooxygenase*; *CYP92A6, typhasterol/6-deoxotyphasterol 2alpha-hydroxylase*; *DET2, steroid 5-alpha-reductase*; *DHDDS, dehydrodolichyl diphosphate synthase*; *DWF4, steroid 22-alpha-hydroxylase*; *DXR, 1-deoxy-d-xylulose 5-phosphate reductoisomerase*; *DXS, 1-deoxy-D-xylulose-5-phosphate synthase*; *FNTA, farnesyltransferase alpha*; *GA20ox, gibberellin 20-oxidase*; *GGPS, geranyl diphosphate synthase*; *HMGCR, 3-hydroxy-3-methylglutaryl-CoA reductase*; *HMGCS, hydroxymethylglutaryl-CoA synthase*; *ispF, 2-C-methyl-D-erythritol 2,4-cyclodiphosphate synthase*; *ispH, 4-hydroxy-3-methylbut-2-en-1-yl diphosphate reductase*; *ispS, isoprene synthase*; *MD, (+)-neomenthol dehydrogenase*; *MVD, diphosphomevalonate decarboxylase*; *NCED, 9-cis-epoxycarotenoid dioxygenase*; *NES1, (3S,6E)-nerolidol synthase*; *lcyB, lycopene beta-cyclase*; *lcyE, lycopene epsilon-cyclase*; *STE24, saccharomyces cerevisiae zinc metallo-protease*; *SQLE, squalene monooxygenase*; *UGT73C, UDP-glucosyltransferase 73C*; *VDE, violaxanthin de-epoxidase*; and *ZDS, zeta-carotene desaturase*.

We detected 3 DSMs, all of which were significantly (*p* < 0.05) increased in SCC needles; among them, the contents of all-trans-heptaprenyl diphosphate, mevalonic acid-5P, and pyruvic acid in SCC needles were 4.05-, 2.95- and 1.17-fold higher than those in WCC needles, respectively (Supplementary Table 12; Figure 7). Twenty-eight candidate DEGs were involved in TBBP, including 14 upregulated unigenes [3 4-hydroxy-3-methylbut-2-en-1-yl diphosphate reductase (ispH), 3 dehydrodolichyl diphosphate synthase (DHDDS), 2 geranyl diphosphate synthase (GGPS), 1 isoprene synthase (ispS), 1 dehydrodolichyl diphosphate synthase (DXR), 1 1-deoxy-D-xylulose-5-phosphate synthase (DXS), 1 hydroxymethylglutaryl-CoA synthase (HMGCS), 1 3-hydroxy-3-methylglutaryl-CoA reductase (HMGCR), and 1 farnesyltransferase alpha (FNTA)] in SCC needles (Supplementary Table 13; Figure 7). Specifically, 3 upregulated genes, i.e., DHDDS, FNTA, and ispS, were located downstream of each branch of TBBP. DXR, DXS, and ispH were located in the 2-methyl-pentaerythritol 4-phosphate (MEP) pathway, and their expressions in SCC needles were 0.94-, 1.98-, and 7.30–11.61-fold higher than those in WCC needles, respectively. HMGCS and HMGCR were located in the mevalonate (MVA) pathway; although they were significantly (*p* < 0.05) upregulated in SCC needles, their expression levels in SCC needles were very low, both <3.40. GGPSs were located in terpenoid synthesis, and their expression in SCC needles was 2.06–19.03-fold those in WCC needles. In addition, 6, 8, 3, 25, 15, and 15 DEGs were involved in mono-, di-, sesqui- and triterpenoid, carotenoid, brassinosteroid, and zeatin biosynthesis, respectively (Supplementary Table 14). Most of these genes involved in brassinosteroid biosynthesis and the di-, sesqui-, and triterpenoid pathways were upregulated in SCC needles. In particular, in the diterpenoid biosynthesis pathway, with the exception of 1 gibberellin 20-oxidase (*GA20ox*), the other 7 unigenes (GA3 and (13E)-labda-7, 13-dien-15-ol synthase) were all downregulated in WCC needles (Supplementary Table 14).

**Figure 8:**
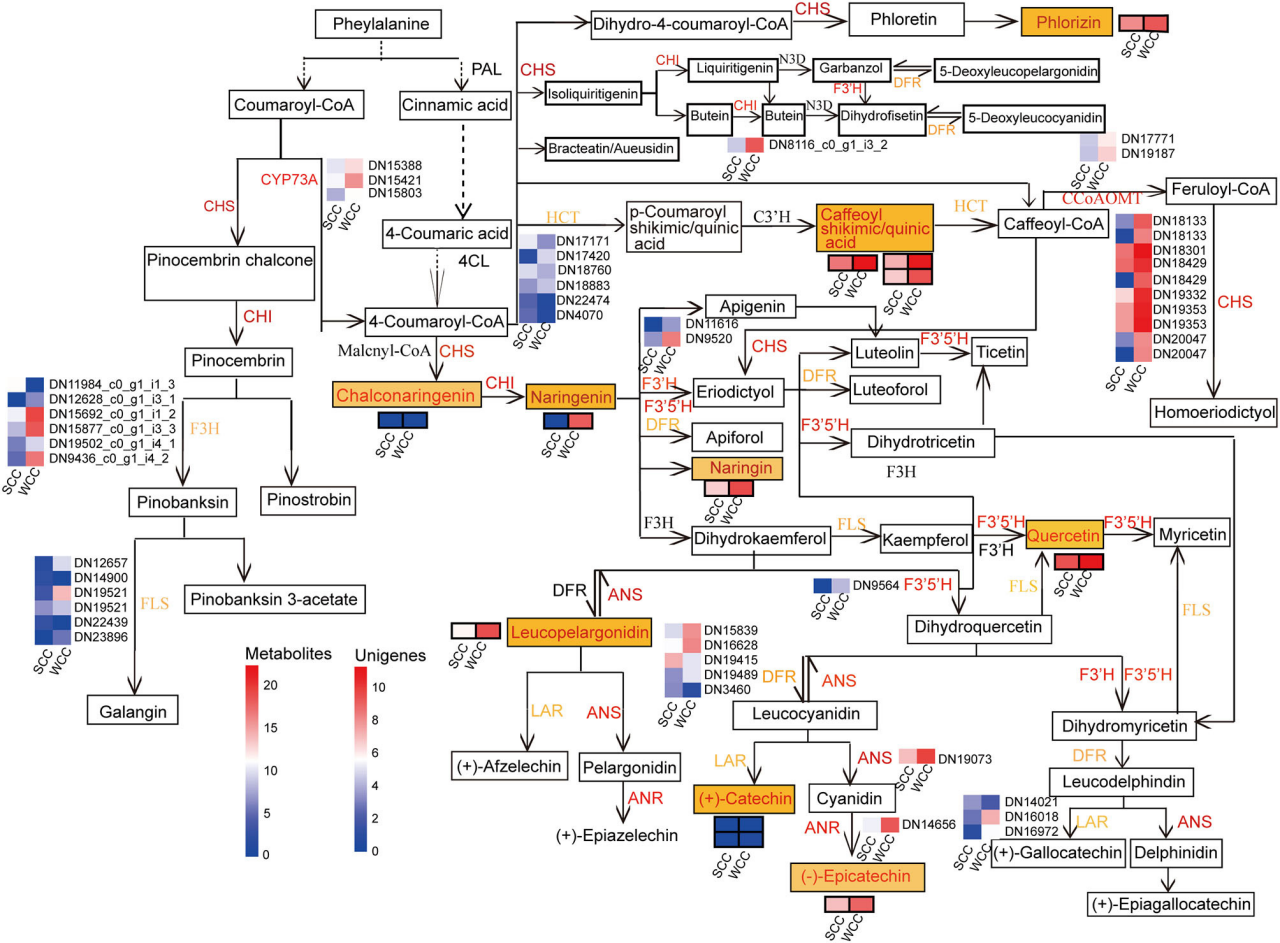
Metabolic pathway map of the flavonoid biosynthesis pathway. SCC and WCC represent summer and winter Chinese cedar needle samples, respectively. Small squares without/with lines indicate the expressed levels of unigenes/metabolites, and red/yellow text indicates up/both up- and downregulated genes or metabolites in WCC needles. The abbreviation of each gene is as follows: A*NR, anthocyanidin reductase gene*; *ANS, anthocyanidin synthase gene*; *CCoAOMT, caffeoyl CoA O-methyltransferase*; *CHI, chalcone isomerase*; *CHS, chalcone synthase*; *CYP73A, cytochrome P450* (*CYP*); *DFR, dihydroflavonol-4-reductase*; *F3H, flavanone 3-hydroxylase*; *F3'H, flavonoid-3'-hydroxylase*; *F3'5'H, flavonoid-3',5'-hydroxylase*; *FLS, flavonol synthase gene*; *HCT, hydroxycinnamoyltransferase*; *LAR, leucoanthocyanidin reductase*.

Forty-seven unigenes were involved in FBP, among which 37 DEGs, including *anthocyanidin reductase and synthase gene* (*ANR*, 1; *ANS*, 1), *chalcone synthase* (*CHS*, 10), *cytochrome P450* (*CYP73A*, 3), *flavonoid-3',5'/3'-hydroxylase* (*F3'5'H*, 1; *F3'H*, 2), *caffeoyl CoA O-methyltransferase* (*CCoAOMT*, 2), and *chalcone isomerase* (*CHI*, 1) genes, and some gene transcripts [*3 dihydroflavonol-4-reductase* (*DFR*), *2 hydroxycinnamoyltransferase* (*HCT*), 5 *flavanone 3-hydroxylase* (*F3H*), 4 *flavonol synthase gene* (*FLS*), and 2 *leucoanthocyanidin reductase* (*LAR*)], were significantly (*p* < 0.05) upregulated in WCC needles (Figure 8; Supplementary Table 15). Correspondingly, 12 metabolites were involved in FBP (Supplementary Table 16; Figure 8). Except for 3 metabolites, which were at low contents in *C. fortunei* needles, the other 9 metabolite contents in WCC needles were 5.66–163.98-fold those in SCC needles (Supplementary Table 16). Specifically, in WCC needles, the *HCT* expressions were 1.17–23.85-fold higher than those in SCC needles, and the contents of caffeoyl shikimic acid and caffeoyl quinic acid were 14.47- and 46.45-fold higher than those in WCC needles. The CHS expressions were 2.85–1276.11-fold in SCC needles, and the contents of chalconaringenin and phlorizin were 41.86- and 5.66-fold in SCC needles. The CHI expression was 26.52-fold higher than that in SCC needles, and the contents of naringenin and naringin were >60-fold higher than those in SCC needles. *F3'5'H, F3'H, F3H*, and *FLS* were expressed at higher levels in WCC needles than those in SCC needles, and the content of quercetin was also significantly increased, which was 8.72-fold higher than that in SCC needles. *DFR* and *LAR* were significantly highly expressed in WCC needles compared to SCC needles, and the contents of leucopelargonidin and catechin were also significantly increased, which were 156.79- and 162.98-fold higher than those in SCC needles, respectively.

### qRT-PCR Validation of DEG Expression

**Figure 9:**
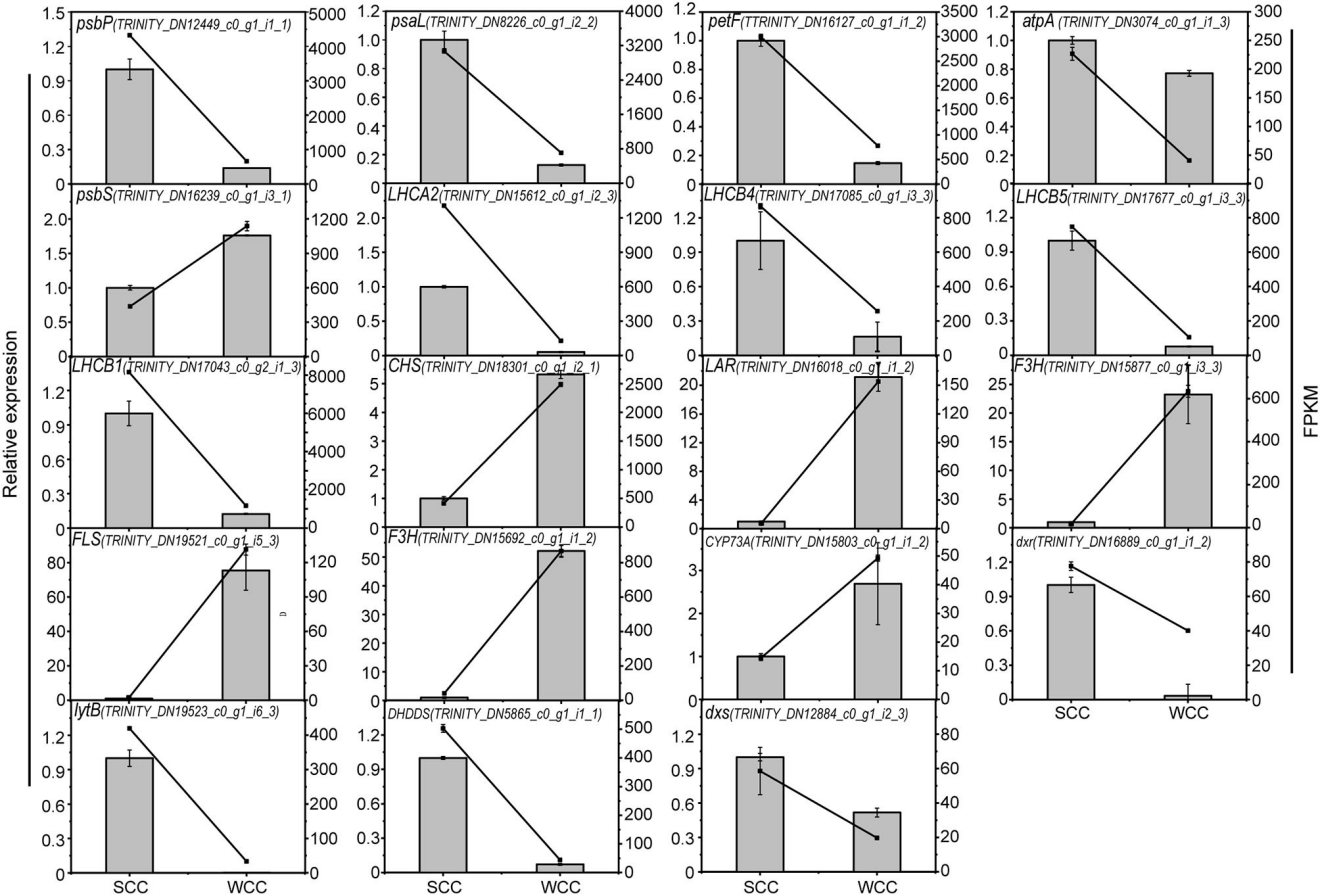
Quantitative real-time polymerase chain reaction (qRT-PCR) validation of select genes involved in the photosynthesis, flavonoid biosynthesis, and terpenoid biosynthesis pathways in *C. fortunei* needles. SCC and WCC represent summer and winter Chinese cedar needle samples, respectively. The line and bar chart represent FPKM and relative expression level, respectively, and each value is the mean ± standard deviation (SD), *n* = 3. FPKM, fragments per kilobase per million. The abbreviation of each gene is as follows: *CHS, chalcone synthase*; *CYP73A, cytochrome P450* (*CYP*); *DHDDS, dehydrodolichyl diphosphate synthase*; *DXR, 1-deoxy-d-xylulose 5-phosphate reductoisomerase*; *DXS, 1-deoxy-D-xylulose-5-phosphate synthase*; *F3H, flavanone 3-hydroxylase*; *FLS, flavonol synthase gene*; F-type ATPase (*atpA*) and light-harvesting chl protein complexes (*Lhca2*, and *Lhcb1, 4, 5*); photosynthetic electron transport *(petF*); photosystem I (*psaL*); photosystem II (*psbP, S*); *ispH, 4-hydroxy-3-methylbut-2-en-1-yl diphosphate reductase*; and *LAR, leucoanthocyanidin reductase*.

To verify the reliability of the transcriptome data, we measured the transcript levels of 18 unigenes by qRT-PCR. The expression patterns of these unigenes were similar to those obtained via RNA-seq analysis (Figure 9). Therefore, our RNA-seq analysis results show high reproducibility and reliability and provide a reference for further research on the key genes involved in photosynthesis, flavonoid, and terpenoid accumulation in the needles of *C. fortunei*.

## Discussion

### Integrated Metabolome and Transcriptome Analysis

Metabolites are intermediate or final products produced during the growth and development of plants, and it is estimated that the number of natural metabolites in plants may be as high as 200,000 (Pichersky and Lewinsohn, 2011). These metabolites are very important not only for the plant itself but also for human energy acquisition and health (De Luca et al., 2012). We detected 3,157 (GC-MS, 277; LC-MS, 2,880) metabolites with clear definitions, among which 519 belonged to the group of DSMs showing the largest differences in the needles of *C. fortunei* between different seasons (Supplementary Figures 2, 5). Previous studies have also examined the differences in the metabolites of *Cryptomeria* but found fewer types. For example, Takada et al. (2004) treated *C. japonica* wood with supercritical water, carried out gel permeation chromatography (GPC), and GC-MS analyses, and detected 31 species expected to be monomer compounds and 22 dimerization products. Xie et al. (2012) used GC-MS to analyze the essential oil of *C. fortunei* samples from different locations and identified 87 compounds, revealing that the chemical differences in the *C. fortunei* needle essential oil samples were attributed to both genetics and the environment and providing a reference for selecting *C. fortunei* needles and essential oils containing important compounds. On the basis of the GC-MS analysis results, we also applied LC-MS and identified more metabolites. The high diversity of these plant metabolites makes them an ideal target for studying the regulatory mechanisms of metabolic biosynthesis and provides a reference for subsequent research.

The “omics” research strategy provides new system-biological insights elucidating the mechanisms of gene expression and metabolite accumulation related to environmental responses. Specifically, plant cells exhibit significant regulatory flexibility by initiating gene expression programs to regulate cell metabolism to adapt to a new environment, thereby responding to environmental changes and enabling the plants to survive under different external environmental conditions. In recent years, extensive research has been conducted on the mechanisms underlying plant metabolite changes, including genetic mechanisms (Meihls et al., 2013; Quadrana et al., 2014). For example, integrated transcriptome and metabolome analyses of tulip trees and ginkgo have revealed important correlations between metabolites and genes in the carotenoid and flavonoid/phenylpropane pathways (Guo et al., 2020; Hao et al., 2020). In this study, our correlation analysis of transcriptome and metabolome profiles showed that the expression levels/contents of some structural genes/metabolites were related to PP/PPP, FBP/RNA transport, flavonoids, terpenoid metabolism, carbon fixation in photosynthetic organisms, and starch and sucrose metabolism (Figure 5; Supplementary Table 10), indicating that gene expression related to the biosynthesis of these compounds is involved in the adaptation of *C. fortunei* to different seasons, but the potential regulatory mechanism and other regulatory characteristics still need followup analysis.

### Impact of SCs on Photosynthesis, Flavonoids, and Terpenoids

In this study, the chloroplast structure of *C. fortunei* needles was shown to be locally damaged in winter, which led to a decrease in the synthesis of chl compounds (Figure 1). This is consistent with our previous study (Zhang et al., 2020) showing that low-temperature stress damages the chloroplast thylakoid membrane structure in needle cells, accelerates the degradation of chl, and affects the photosynthetic reaction rate in winter. Accordingly, we found that DEGs were enriched in PP (Figure 4). In WCC needles, most of the genes encoding the photosystem subunits, LHCI and LHCII, including chl-binding proteins, ATP synthase, cytochrome b6, and ferredoxins in the electron transport chain showed reduced expression (Figure 6). *Lhca1–4* and *Lhcb1–7*, which are involved in PPP, were downregulated (Figure 6), showing that SCs have a serious impact on the stability of the photosynthetic system LHCII in the needles of *C. fortunei*. It is worth noting that *PsbA* and *PsbS* were upregulated in WCC needles (Figure 6). It is generally believed that plant PSII, composed of more than 25 subunit proteins, plays a key role in the damage caused by multiple stresses; among these proteins, the D1 protein encoded by chloroplast *psbA* is the target of many types of stress damage (Yamamoto et al., 2008). The degradation of the damaged D1 protein and the rapid synthesis of a new D1 protein are key steps in PSII functional repair; therefore, the timely synthesis of the D1 protein plays an extremely important role in the rapid turnover of the D1 protein under stress (Liu et al., 2006; Yoshioka et al., 2006). The high expression of *psbA* in winter is beneficial to the repair of D1 under low-temperature stress and the improvement of photosynthetic efficiency. In addition, under winter light conditions, the pH value within the plant thylakoid cavity will decrease, thereby activating the photoprotective protein, *PsbS* embedded in the thylakoid membrane, in turn inducing the very effective high-light protection mechanism of the energy-dependent quenching. This allows for plants to safely dissipate the excess light energy absorbed by the light-harvesting complex in the form of heat, thereby reducing or avoiding photooxidative damage (Nield et al., 2000).

**Figure 10:**
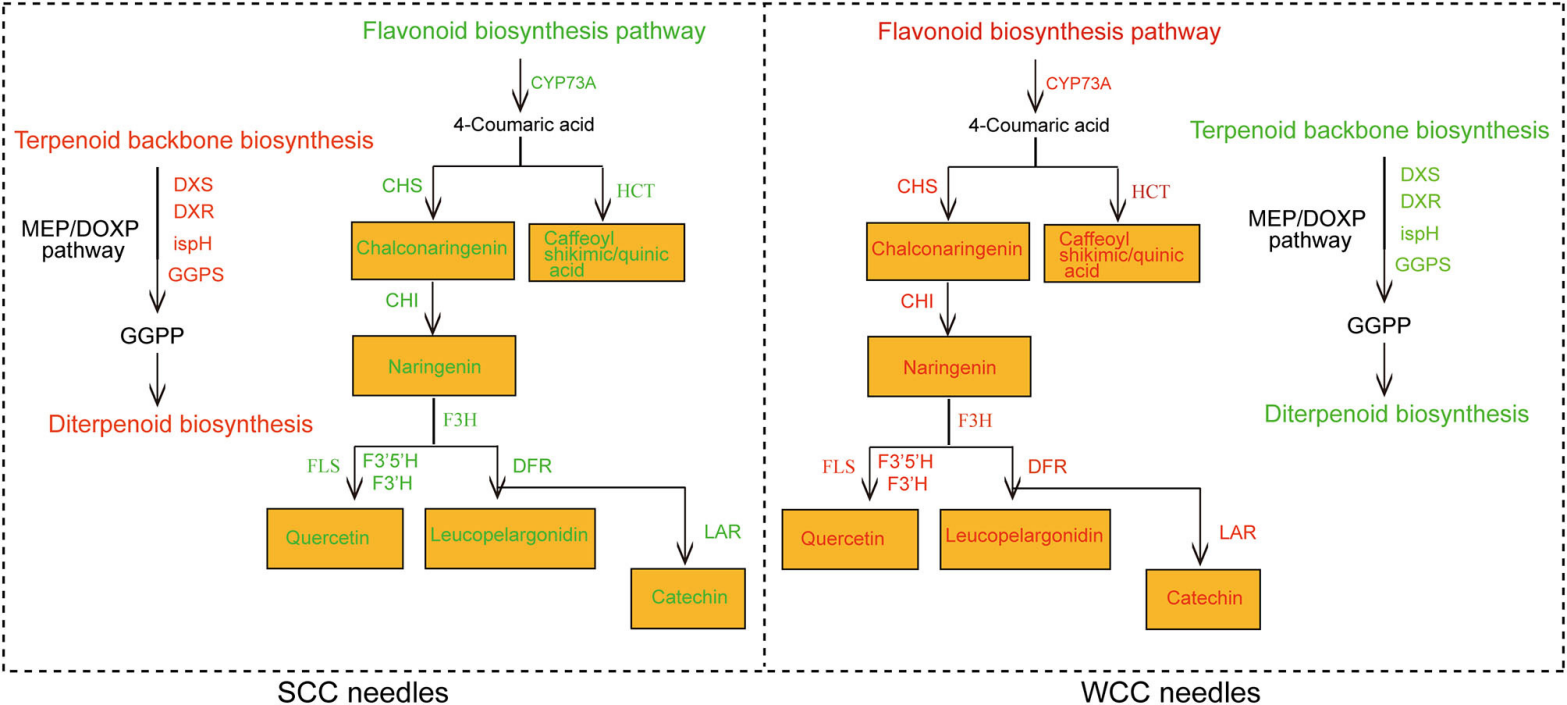
Regulatory network and mechanism of terpenoid and flavonoid biosynthesis in *C. fortunei* needles. SCC and WCC represent summer and winter Chinese cedar needle samples, respectively. Green text indicates downregulated metabolites/genes/metabolic pathways, and red text indicates upregulated metabolites/genes/metabolic pathways. The abbreviation of each gene is as follows: *CHI, chalcone isomerase*; *CHS, chalcone synthase*; *CYP73A, cytochrome P450* (*CYP*); *DFR, dihydroflavonol-4-reductase*; *DXR, 1-deoxy-d-xylulose 5-phosphate reductoisomerase*; *DXS, 1-deoxy-D-xylulose-5-phosphate synthase*; *F3H, flavanone 3-hydroxylase*; *F3'H, flavonoid-3'-hydroxylase*; *F3'5'H, flavonoid-3',5'-hydroxylase*; *FLS, flavonol synthase gene*; *GGPS, geranyl diphosphate synthase*; *HCT, hydroxycinnamoyltransferase*; *ispH, 4-hydroxy-3-methylbut-2-en-1-yl diphosphate reductase*; and *LAR, leucoanthocyanidin reductase*.

Flavonoids are secondary metabolites in the phenylpropanoid pathway, within which coumarin-coenzyme A is the precursor of FBP, which acts as a bridge between primary and secondary metabolism (Thomas, 2010; Loke et al., 2017). Flavonoids are a group of polyphenol secondary metabolites that have a variety of biological functions, such as roles in plant tissue coloration, photoprotection, abiotic stress, tropism, and other processes related to growth development and stress responses (Brown et al., 2001; Buer et al., 2010; Saito and Matsuda, 2010). In this study, flavonoids were found to accumulate under low-temperature (winter climate) conditions (Figure 8). Similar observations have been made in plants that turn yellow in autumn and winter, such as ginkgo (Cheng et al., 2012), in which low temperature increases the content of flavonoids in plants in autumn and winter. At the same time, we found that most genes in the flavonoid pathway, such as *ANR, ANS, CHS, CYP73A, F3'H*, and *CHI*, were upregulated in WCC needles (Figure 8), which is consistent with the results of previous studies (Liu et al., 2015; Song et al., 2017) showing that an increase in FBP gene expression levels leads to a greater accumulation of flavonoids. Through integrated transcriptome and metabolome analysis, we identified key genes in FBP, that is, *HCT, CHS, CHI, F3H, F3'H, F3'5'H, FLS, DFR*, and *LAR*, that regulate the differential synthesis of flavonoids in *C. fortunei* needles across different seasons (Figures 8, 9). In WCC needles, *HCT* expression was high, and the content of chlorogenic acid (caffeoyl shikimic acid and caffeoyl quinic acid) was increased (Supplementary Tables 15, 16). *HCT* catalyzes the synthesis of acetyltransferase to generate p-coumaroylquinic acid, which is then hydroxylated by 3'-p-coumaroyltransferase (*C3'H*) to generate chlorogenic acid (Hoffmann et al., 2003). Studies in a variety of plants have found that *HCT* promotes chlorogenic acid synthesis (Payyavula et al., 2015; Zhang et al., 2018; Chen et al., 2020). Therefore, we believe that *HCT* promotes chlorogenic acid synthesis in WCC needles (Figure 10). In WCC needles, *CHS* was highly expressed, and the content of chalconaringenin was increased; *CHI* was highly expressed, and the content of naringenin and naringin were increased (Supplementary Tables 15, 16). *CHS*, the first key enzyme that directs the phenylpropanoid metabolic pathway to FBP, catalyzes the formation of chalconaringenin from p-coumaroyl-CoA and malonyl-CoA (Austin and Joseph, 2003) and drives the upstream compounds in the phenylpropane synthesis pathway to flow to the FBP branches (Winkel-Shirley, 2001; Grotewold, 2006), thereby upregulating the overall production of flavonoid metabolites. *OsCHS24* and *OsCHS8* catalyze the formation of chalconaringenin from p-coumaroyl-CoA and malonyl-CoA in rice (Jiang et al., 2006; Park et al., 2020) identified the *in vitro* enzymatic reaction products of moss (*Physcomitrella patens*) *CHS* by radio thin layer chromatography and found that the main product was chalconaringenin. Therefore, we believe that *CHS* promotes the synthesis of chalconaringenin in WCC needles and simultaneously upregulates genes/metabolites in downstream pathways (Figure 10). Chalconaringenin is spontaneously isomerized by *CHI* to form naringenin (Dixon and Paiva, 1995), which guarantees the synthesis of flavonols, flavanones, anthocyanin glycosides, and other flavonoids. Therefore, we believe that the high content of chalconaringenin in WCC needles generates a high content of naringenin under the action of the *CHI* enzyme (Figure 10). The key enzymes of flavonol biosynthesis (*FLS, F3H, F3'H*, and *F3'5'H*) were expressed at high levels in WCC needles, and the quercetin content was also upregulated (Supplementary Tables 15, 16). Naringenin is catalyzed by *F3H* to form dihydrokaempferol, which is catalyzed by *FLS* to form kaempferol, and *F3'H* and *F3'5'H* catalyze the hydroxylation of the 3'and 5' positions of the flavanone B ring to generate flavanones (quercetin), dihydroflavonols, and flavonols with different degrees of hydroxylation (Winkel-Shirley, 2001). The flowers of f3h mutants of zinnia (*Zinnia elagans*) are white, and flavonols and anthocyanins are rarely detected in these plants (Forkmann and Stotz, 1984). Overexpression of maize (*Zea mays*) *ZmFLS1* in *Arabidopsis* fls1 mutants partially restored the phenotype caused by flavonol depletion (Falcone Ferreyra et al., 2010); *NtFLS* in transgenic tobacco (*Nicotiana tabacum* cv Xanthi) plants showed a 25–93% reduction in quercetin content (Mahajan et al., 2011, 2012). Heterologous expression of grapevine (*Vitis vinifera*) *VvF3'H* in the petunia ht1 mutant showed that the major flavonol in flowers of transgenic plants changed from kaempferol to quercetin (Bogs et al., 2006). Apple (*Malus* × *domestica*) *MdF3'HI* and *MdF3'HIIb* were overexpressed in tobacco and *Arabidopsis*, respectively, and the kaempferol content in transgenic *Arabidopsis* seedlings and tobacco flowers was significantly decreased, while the quercetin content was significantly increased (Han et al., 2010). Therefore, we believe that the upregulation of flavonoid upstream genes and key enzyme genes of flavonol biosynthesis promotes the upregulation of quercetin in WCC needles (Figure 10). *DFR* and *LAR* were upregulated in WCC needles, as were the contents of leucopelargonidin and catechin (Supplementary Tables 15, 16). *DFR* is a key enzyme in the formation step of flavonoids, which reduces dihydroflavonols to leucoanthocyanidins, and leucoanthocyanidins are directly reduced by *LAR* to obtain nonphenotypic catechins. Studies on tea trees found that the *DFR*/*LAR* two-step reaction is the key step in catalyzing the formation of catechins, and *DFR* is the rate-limiting step (Punyasiri et al., 2004). During the leaf development of different tea varieties, the expression of the *DFR* gene was significantly correlated with the accumulation of total catechins (Mamati et al., 2006). Stafford et al. (1985) used *DFR* in a crude enzyme solution to convert dihydroflavonols into leucoanthocyanidins, which are further converted into catechins under the catalysis of *LAR*. The leaf color regulatory gene from maize was transferred into apple plants, and the results showed that the expression level of *LAR* in transgenic plants was significantly increased and that catechins accumulated in large amounts (Li et al., 2007). Therefore, we believe that the high expression of *DFR* and *LAR* in WCC needles promoted the accumulation of leucoanthocyanins and catechins (Figure 10). The metabolic network of this pathway is complex and includes many branches; therefore, the cooperative expression and regulatory mechanism of each gene need to be further studied.

In this study, in SCC needles, metabolites in TBBP were increased, and *HMGCS, HMGCR, DXS, DXR, ispH*, and *GGPS* were upregulated (Figure 7; Supplementary Tables 13, 14). There are two pathways, i.e., cytoplasmic MVA and plastid MEP, for TBBP (Estevez et al., 2001; Friesen and Rodwell, 2004). The expression of *HMGCS* and *HMGCR* genes was low in *C. fortunei* needles (Figure 7; Supplementary Table 14), possibly because genes in the MVA pathway were highly expressed mainly in the roots and shoots (Chen et al., 2015). *HMGCS* and *HMGCR* catalyze acetyl-CoA to mevalonate, a key rate-limiting reaction in the MVA pathway (Rodríguez-Concepción and Boronat, 2002; Rohmer, 2007), and positively regulate the synthesis of terpenoids (Gondet et al., 1992; Zhang et al., 2019). Sesquiterpenoids, diterpenoids, phytosterols, brassinolides, and cytokinins are mainly synthesized in the cytoplasm through the MVA pathway (Dubey et al., 2003). Therefore, the low expression of these two genes in SCC needles slightly increased the synthesis of sesquiterpenoids and diterpenoids in *C. fortunei*. The expression of *DXS, DXR*, and *ispH* genes and the content of metabolite mevalonic acid-5P in the MEP pathway were at high levels in SCC needles (Figure 7; Supplementary Tables 13, 14). In higher plants, the MEP pathway is predominant (Piel et al., 1998). This pathway operates within the chloroplast, so these genes are highly expressed in needles, especially in SCC needles (Chen et al., 2015). *DXS, DXR*, and *ispH* are key rate-limiting factors in the MEP pathway and help synthesize the terpenoid precursor IPP and its isomer, DMAPP (Lange et al., 1998; Takahashi et al., 1998). These genes promote the synthesis of hemi-, mono-, di-, and tetraterpenoids, as well as the synthesis of isoprenoids bound to chloroplasts (β-carotene, lutein, isoprene chain of chl, and plastoquinone), through the MEP pathway (Lichtenthaler et al., 1997). We found that *GGPS* was highly expressed in SCC needles compared with WCC needles (Figure 7; Supplementary Table 14). *GGPS* utilizes an allylic substrate and IPP to synthesize GGPP. The GGPP is not only the most direct precursor of carotenoid biosynthesis but also the precursor of gibberellin, chl, and plastoquinone in plants. Therefore, the highly expressed *GGPS* in SCC needles promotes the synthesis of terpenoids, especially diterpenoids (Figure 10). In SCC needles, *CYP701* (*GA3*), in the diterpenoid pathway, was upregulated, which increased the expression of the downstream metabolite GA12-aldehyde, whereas *GA20ox* was downregulated, leading to less accumulation of GA53, 9, and 20 (Figure 7). We believe this is similar to previous observations that only a few GA molecules (e.g., GA1, 3, 4, and 7) show biological activity in plants (Spielmeyer et al., 2002; Li et al., 2014); GA53 accumulates less in plants, while the levels of its main products, GA20 and GA1 increase. This suggests that the metabolic activity of *C. fortunei* needles is high in SCC needles; therefore, the trees grow faster.

## Conclusion

In this study, we obtained 519 DSMs and 14,057 DEGs through transcriptomic and metabolomic analyses, indicating that metabolic reorganization and changes in transcript expression levels were triggered by the seasonal transition. We found that SCs have the greatest impact on P(P)P, followed by TBBP and FBP. Through integrated transcriptomic and metabolomic analyses, *DXS, DXR, ispH*, and *GGPS* in the MEP pathway of TBBP were highly expressed in SCC needles, promoting the accumulation of terpenoids, especially diterpenoids; conversely, 9 genes (*HCT, CHS, CHI, F3H, F3'H, F3'5'H, FLS, DFR*, and *LAR*) related to FBP were highly expressed in WCC needles, promoting flavonoid accumulation (Figure 10). This study broadens the understanding of the effects of SCs on the metabolites of *C. fortunei* needles and improves the understanding of metabolite regulatory systems in plant tissues under SCs. This provides the basis for the timely extraction of plant metabolites and provides an important reference for the genetic improvement of plants rich in terpenoids and flavonoids. Future research needs to clarify the precise functions of specific genes in needles in different seasons; it is also necessary to further determine how specific factors, such as temperature or light (e.g., photoperiod and light intensity), participate in the regulation of photosynthesis and terpenoid and flavonoid metabolism.

## Data Availability Statement

Raw reads have been deposited as a BioProject under accessions PRJNA793065 (SUB10872191) and PRJNA697258 (SUB9488615 [SAMN17672905-07)] and metabolomics data can be found at MetaboLights under number MTBLS4099.

## Author Contributions

JX conceived and designed the experiments. YZ performed the experiments. JY, HH, GW, and JC analyzed the data. LY and YZ wrote the manuscript. All authors read and approved the final manuscript.

## Funding

This work was supported by grants from the Fujian Province Science and Technology Research Funding for the Fourth Tree Breeding Program of Chinese fir (Min Lin Ke 2016-35), Seed Industry Innovation and Industrialization Project of Fujian Province (ZYCX-LY-202101), the Postgraduate Research & Practice Innovation Program of Jiangsu Province (KYCX21_0918), and the Priority Academic Program Development of Jiangsu Higher Education Institutions (PAPD).

## Conflict of Interest

The authors declare that the research was conducted in the absence of any commercial or financial relationships that could be construed as a potential conflict of interest.

## Publisher's Note

All claims expressed in this article are solely those of the authors and do not necessarily represent those of their affiliated organizations, or those of the publisher, the editors and the reviewers. Any product that may be evaluated in this article, or claim that may be made by its manufacturer, is not guaranteed or endorsed by the publisher.

## Abbreviations

*CHI*: *Chalcone Isomerase*
chl: Chlorophyll
CHS: Chalcone Synthase
DEGs: Differentially Expressed Unigenes
*DFR*: *Dihydroflavonol-4-Reductase*
DSMs: Differentially Synthesized Metabolites
*DXR*: *1-Deoxy-D-Xylulose 5-Phosphate Reductoisomerase*
*DXS*: *1-Deoxy-D-Xylulose-5-Phosphate Synthase*
*F3H*: *Flavanone 3-Hydroxylase*
*F3'H*: *Flavonoid-3'-Hydroxylase*
*F3'5'H, Flavonoid-3'*: *5'-Hydroxylase*
FBP: Flavonoid Biosynthesis Pathway
*FLS*: *Flavonol Synthase Gene*
GC-MS: Gas Chromatography–Mass Spectrometry
*GGPS*: *Geranyl Diphosphate Synthase*
GO: Gene Ontology
*HCT*: *Hydroxycinnamoyltransferase*
*HMGCR*: *3-Hydroxy-3-Methylglutaryl-CoA Reductase*
*HMGCS*: *Hydroxymethylglutaryl-CoA Synthase*
*ispH*: *4-Hydroxy-3-Methylbut-2-En-1-Yl Diphosphate Reductase*
KEGG: Kyoto Encyclopedia of Genes and Genomes
*LAR*: *Leucoanthocyanidin Reductase*
LC-MS: Liquid Chromatography-Mass Spectrometry
*MEP*: *2-Methyl-Pentaerythritol 4-Phosphate*
MVA: Mevalonate
OPLS-DA: Orthogonal Partial Least Squares-Discriminant Analysis
PP: Photosynthesis Pathway
PPP: Photosynthesis-Antenna Proteins Pathway
PS: Photosystem
qRT-PCR: Quantitative Real-Time Polymerase Chain Reaction
SC: Seasonal Change
TBBP: Terpenoid Backbone Biosynthesis Pathway.
