# Supplementary material for: Transcriptome and Metabolome Analyses Reveal Differences in Terpenoid and Flavonoid Biosynthesis in Cryptomeria fortunei Needles Across Different Seasons
Source: Front Plant Sci. 2022 Jul 22;13:862746. doi: 10.3389/fpls.2022.862746 (PMC9355645; doi:10.3389/fpls.2022.862746)
Supplement: Supplementary file 1 [file Data_Sheet_1.docx]

Supplementary Material

Supplementary Figures

**
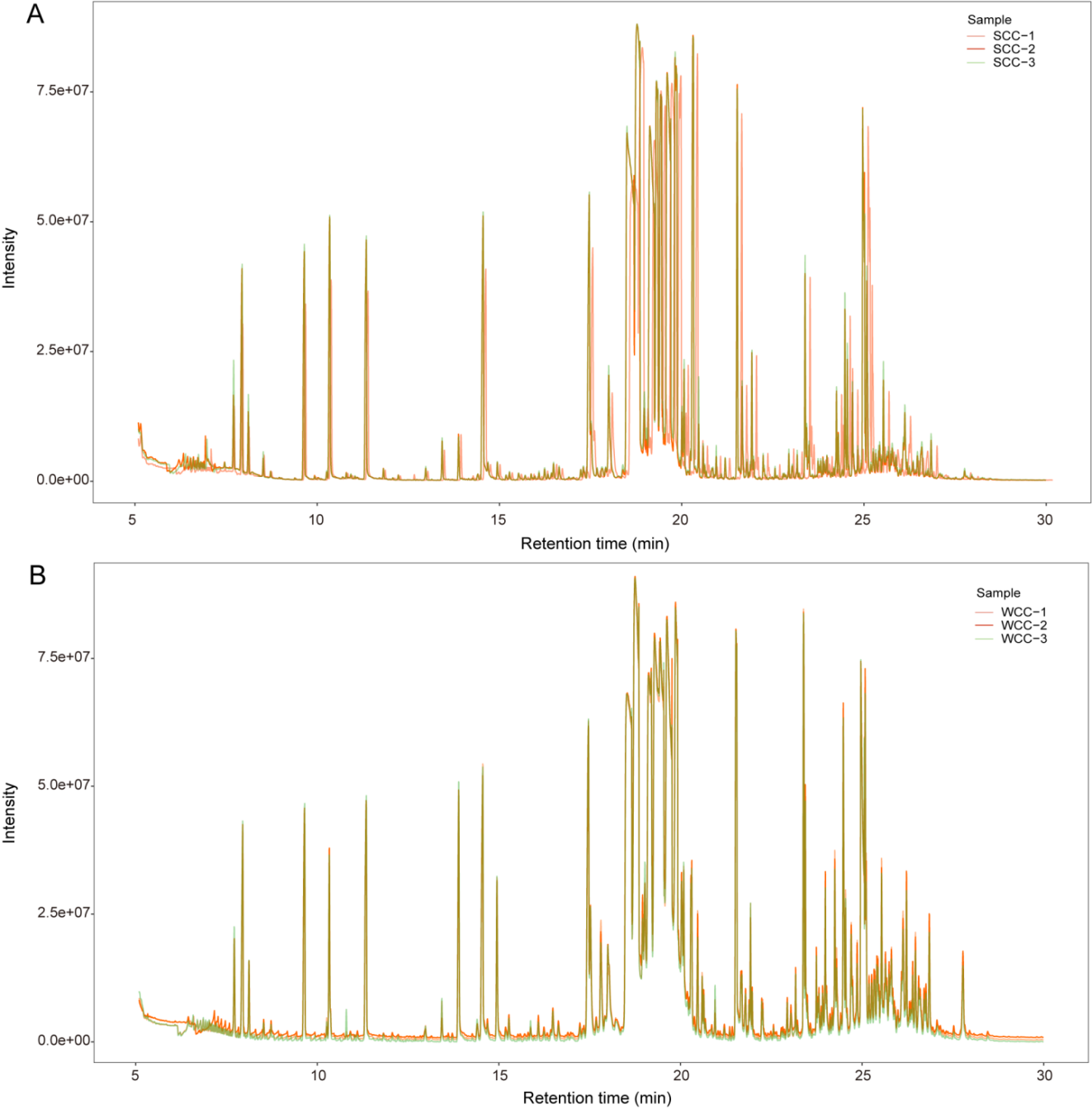
Supplementary Figure 1.** Total ion chromatogram. Total ion chromatogram of SCC **(A)** and WCC **(B)**. SCC and WCC, summer and winter Chinese cedar needle samples, respectively. In each panel, each sample is displayed in a separate color and corresponds to 3 biological repeats (n = 3). The *x*-axis represents retention time, and the *y*-axis represents intensity.


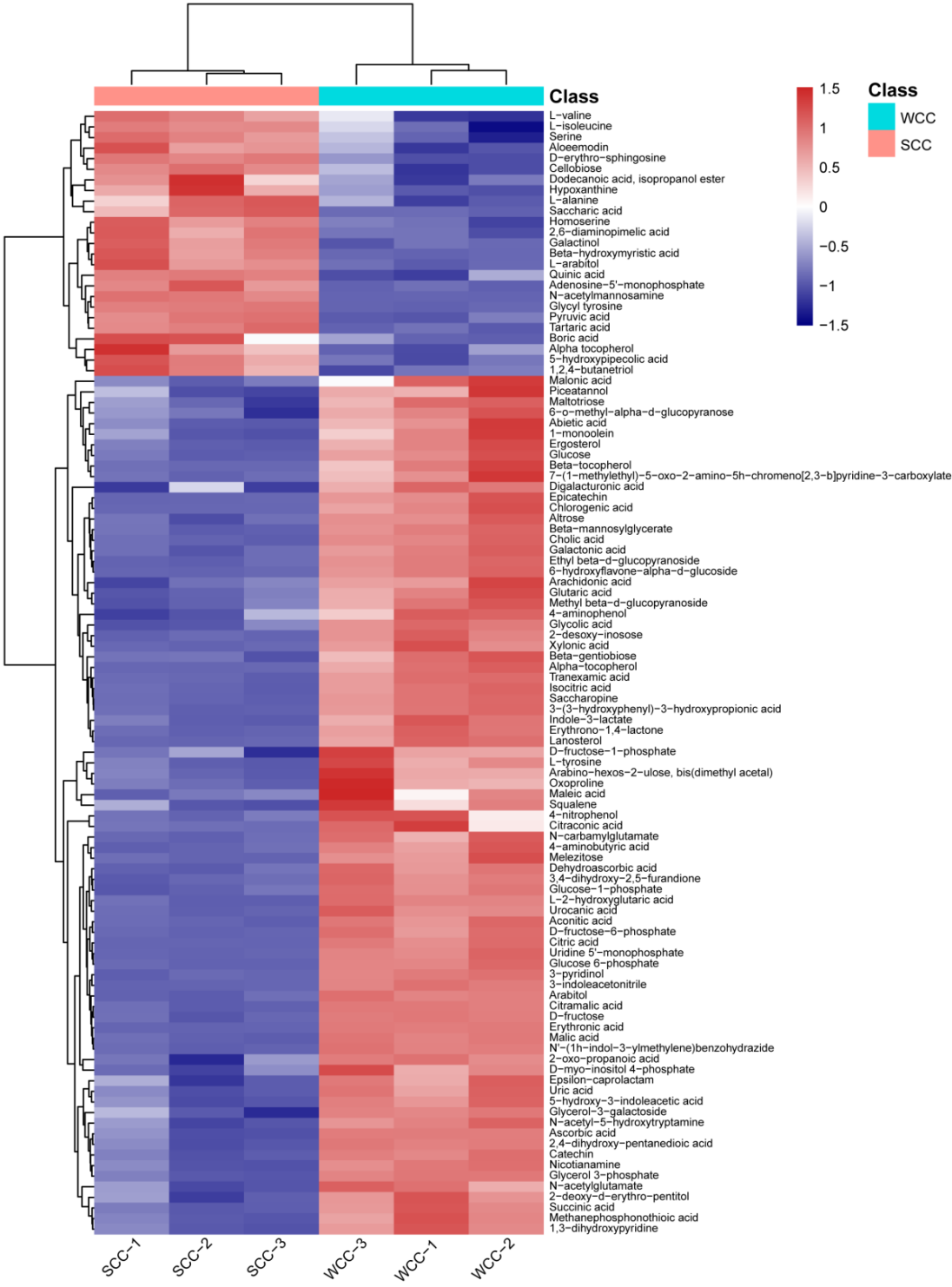


**Supplementary Figure 2.** Hierarchical clustering heatmap of differentially synthesized metabolites (DSMs) identified by GC-MS. Each column and row represent a sample and a DSM, respectively, and the colors indicate the expression levels of DSMs. SCC and WCC, summer and winter Chinese cedar needle samples, respectively, and SCC_1, SCC_2, and SCC_3 represent 3 repetitions.


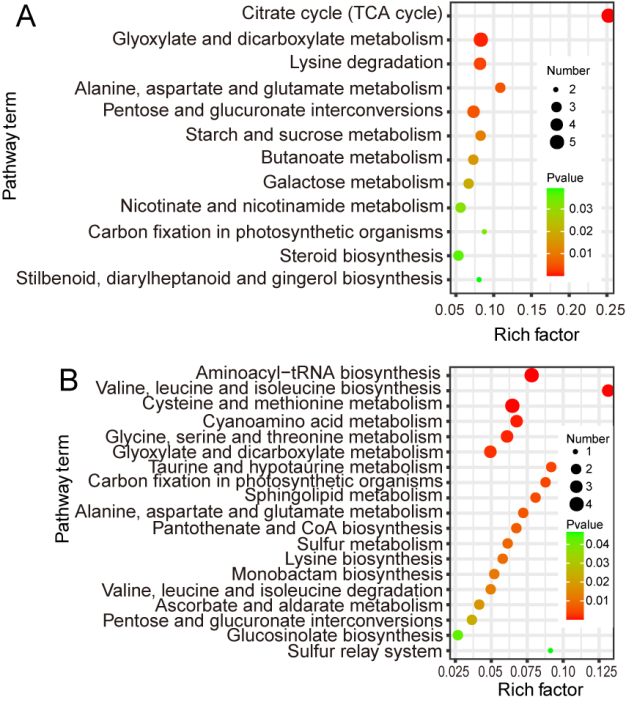


**Supplementary Figure 3.** KEGG analysis of differentially synthesized metabolites (DSMs) identified by GC-MS. **(A)** KEGG analysis of upregulated DSMs in WCC; **(B)** KEGG analysis of downregulated DSMs in WCC. The *x*- and *y*-axes represent the enrichment factor and pathway term, respectively. The colors and sizes of the dots represent the significance (*p*) and number of metabolites, respectively.

**
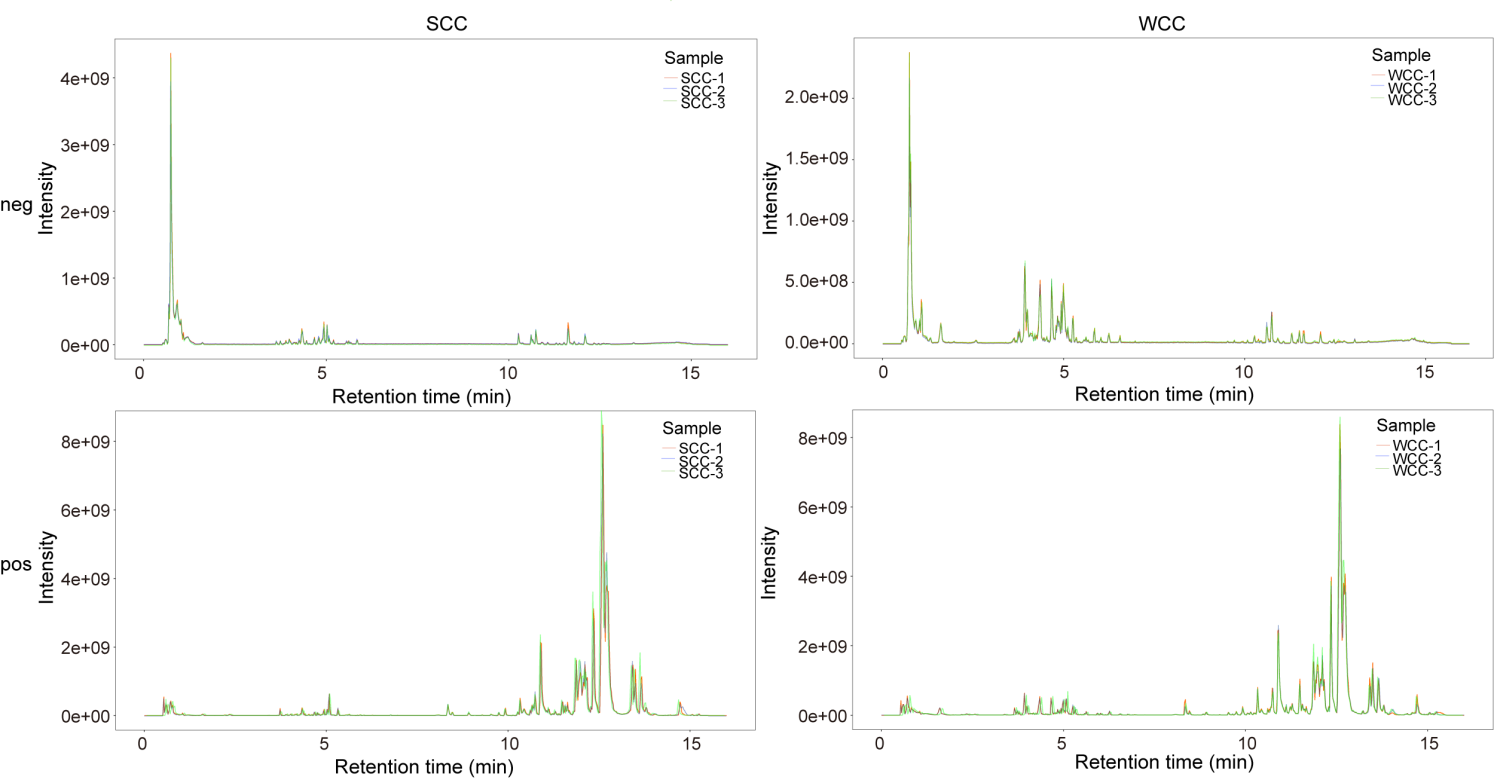
Supplementary Figure 4.** Needle metabolite total ion chromatograms obtained in positive or negative ion mode. The *x*-axis represents retention time, and the *y*-axis represents intensity. SCC and WCC, summer and winter Chinese cedar needle samples, respectively. In each panel, the sample names SCC_1, SCC_2, and SCC_3 indicate 3 biological repeats (n = 3), each displayed in a separate color.

**
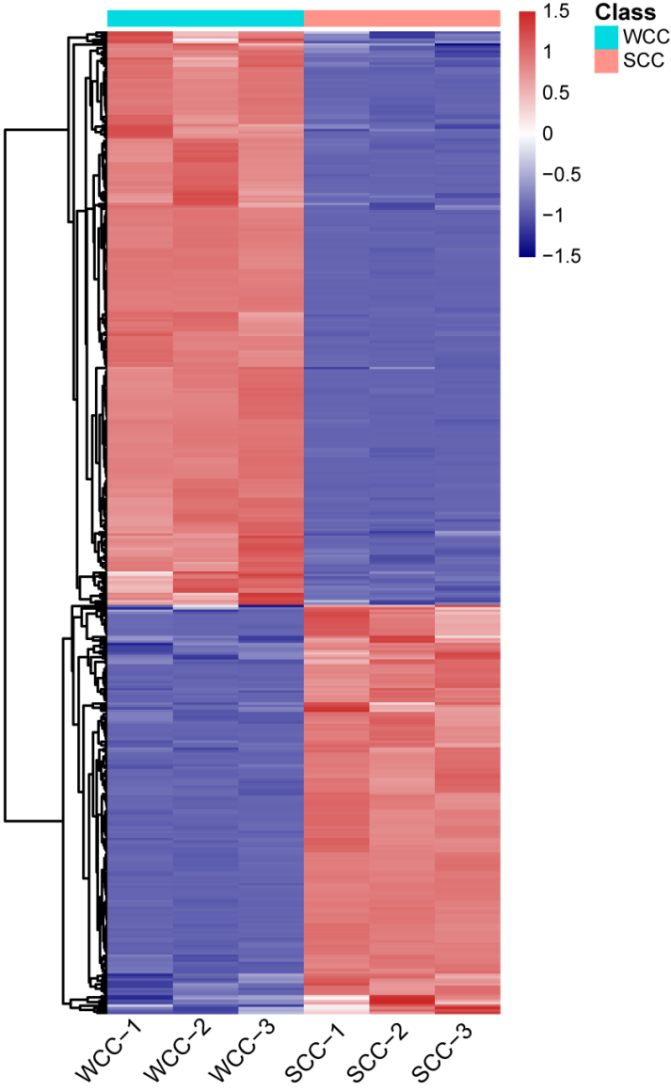
Supplementary Figure 5.** Hierarchical clustering heatmap of differentially synthesized metabolites (DSMs) identified by LC-MS. Each column and row represent a sample and a DSM, respectively, and the colors indicate the expression levels of DSMs. SCC and WCC, summer and winter Chinese cedar needle samples, respectively, and SCC_1, SCC_2, and SCC_3 represent 3 repetitions.


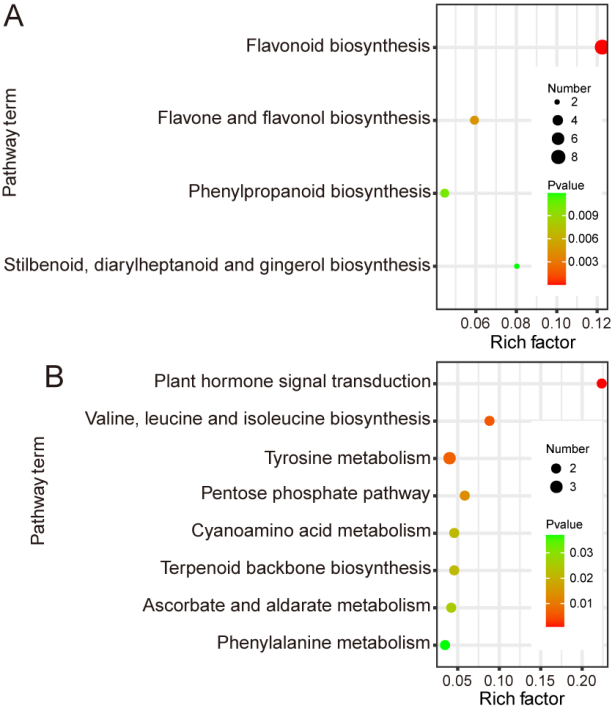
**Supplementary Figure 6.** KEGG analysis of differentially synthesized metabolites (DSMs) identified by LC-MS. **(A)** KEGG analysis of upregulated DSMs in WCC; **(B)** KEGG analysis of downregulated DSMs in WCC. The *x*- and *y*-axes represent the enrichment factor and the pathway term, respectively. The colors and sizes of the dots represent the significance (*p*) and the number of metabolites, respectively.


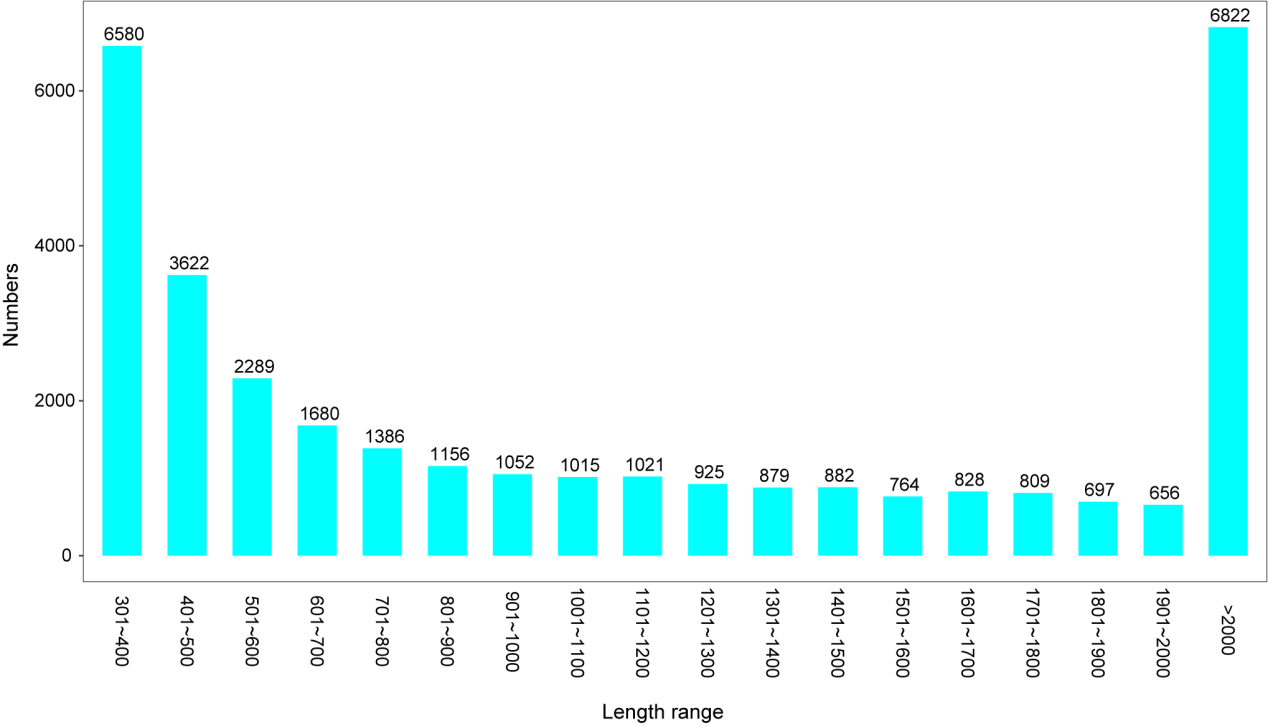
**Supplementary Figure 7.** Length distribution plot of unigenes. The *x*- and *y*-axes represent the length range and corresponding number for each length, respectively.


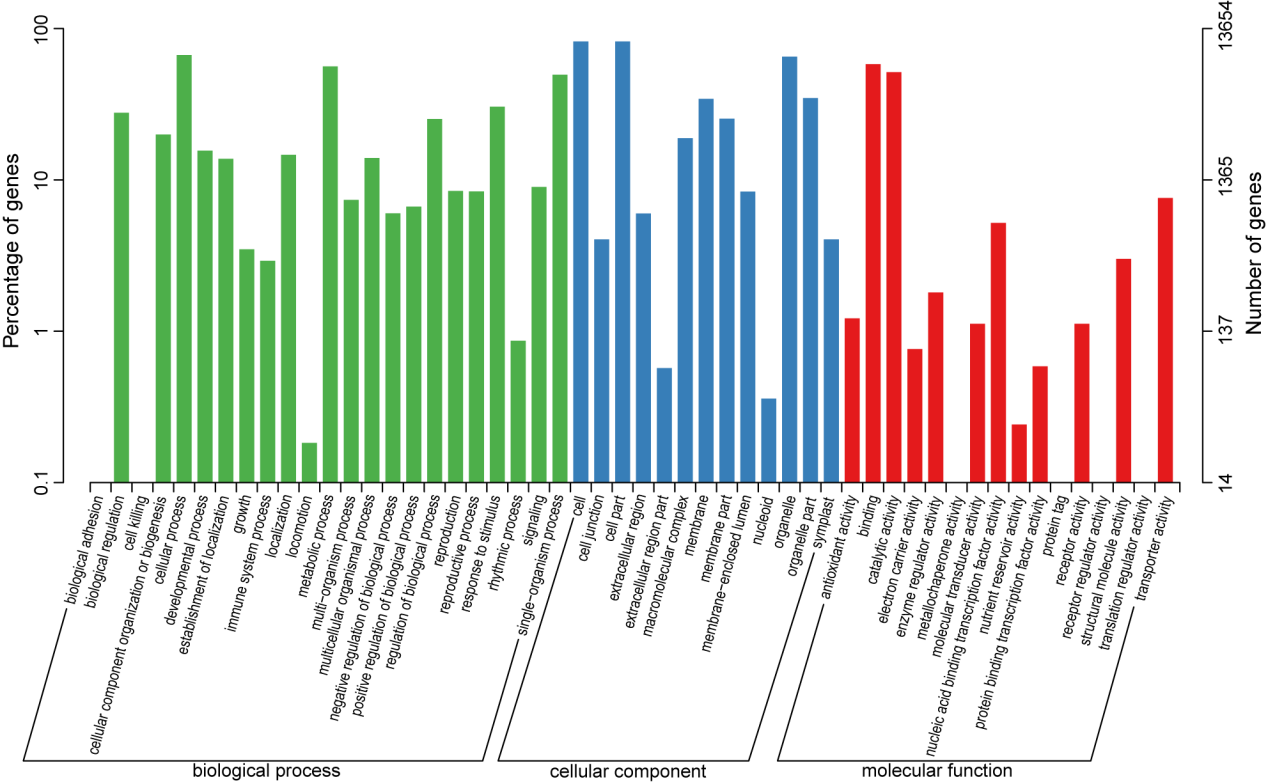
**Supplementary Figure 8.** GO analysis. The *x*-axis represents the GO functional classification, the *y*-axis on the left represents the percentage of genes annotated to this class, and the *y*-axis on the right represents the number of unigenes.


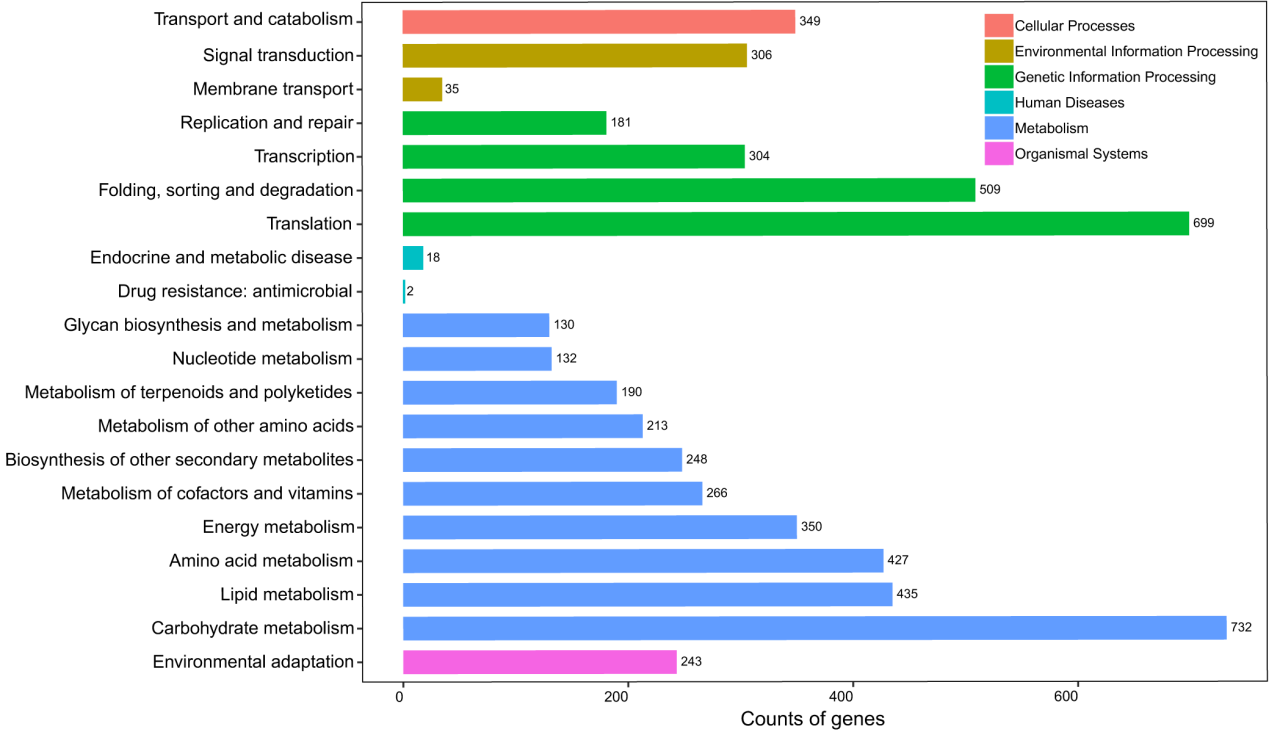
**Supplementary Figure 9.** KEGG annotation statistics. The *x*-axis represents the number of genes, the *y*-axis represents the name of the pathway, and the number on the right side of the column represents the number of genes annotated to the pathway.


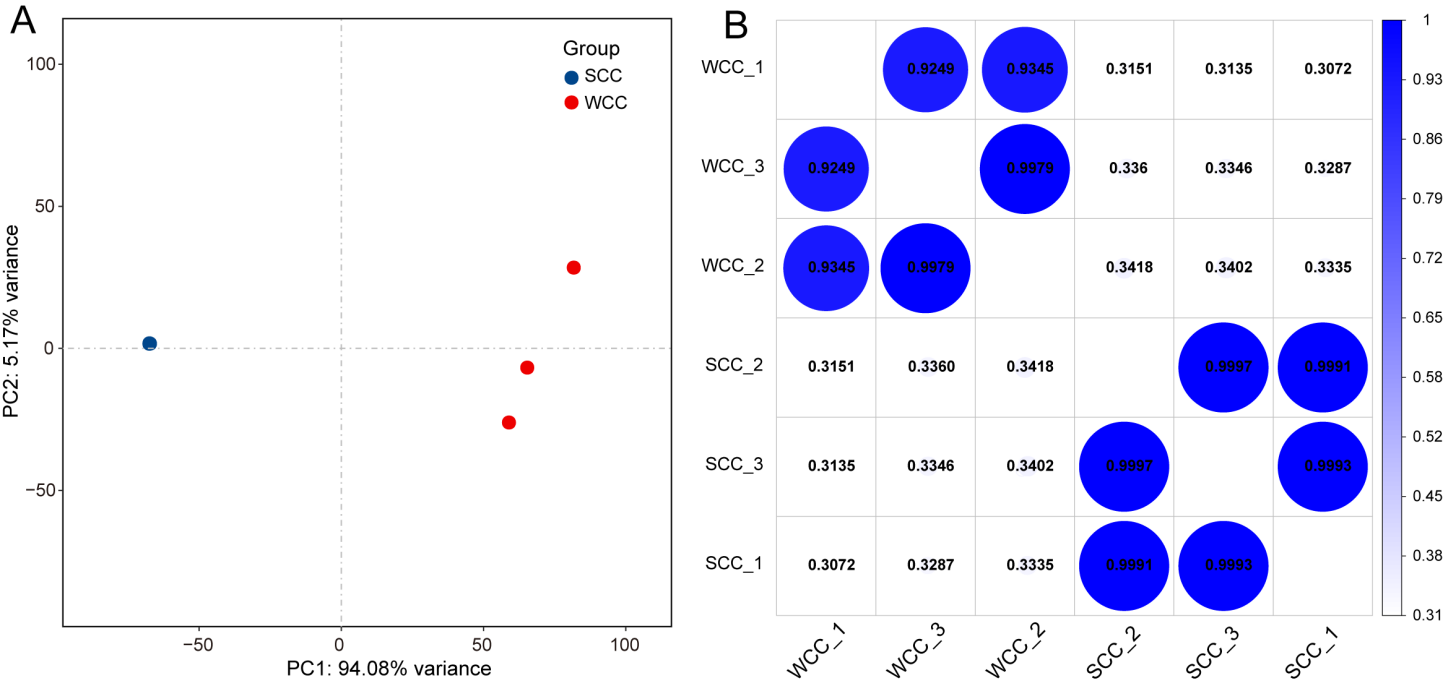
**Supplementary Figure 10.** Principal component analysis and correlation test between samples. **(A)** Principal component analysis. The *x*- and *y*-axes represent the first (PC1) and second (PC2) principal components, respectively. **(B)** Intersample correlation coefficient heatmap. The *x*- and *y*-axes represent the sample name, and the color represents the size of the correlation coefficient. SCC and WCC are the summer and winter Chinese cedar needle samples, respectively, and SCC_1, SCC_2, and SCC_3 represent 3 repetitions.

**Supplementary Table 1.** Sequencing data quality preprocessing results.

| **Sample** | **Raw bases (G)** | **Clean bases (G)** | **Valid bases (%)** | **Q30 (%)** | **GC (%)** |
| --- | --- | --- | --- | --- | --- |
| SCC_1 | 6.61 | 6.38 | 96.56 | 96.32 | 44.87 |
| SCC_2 | 7.54 | 7.26 | 96.32 | 96.31 | 45.00 |
| SCC_3 | 6.55 | 6.35 | 96.86 | 96.40 | 44.97 |
| WCC_1 | 7.83 | 7.44 | 95.03 | 95.69 | 44.74 |
| WCC_2 | 7.17 | 6.85 | 95.47 | 95.36 | 44.20 |
| WCC_3 | 7.32 | 6.89 | 94.10 | 95.60 | 44.26 |

**Supplementary Table 2.** All unigene primers used in this study.

| **Unigene** | **Gene name** | **Forward primer (5’-3’)** | **Reverse primer (5’-3’)** |
| --- | --- | --- | --- |
| TRINITY_DN12449_c0_g1_i1_1 | *psbP* | GGATTTGACACCAATGCGGTAG | GCTGAGATGAGTTGATGTTTCCCT |
| TRINITY_DN8226_c0_g1_i2_2 | *psaL* | AAGCCCACATTTCAAGTGATAGA | AGAGGTGCGATAAGCAGGTAGA |
| TRINITY_DN16127_c0_g1_i1_2 | *petF* | CTGCCTTACTCTTGCCGTTCT | GCCACTCCCAATCTGGTCAT |
| TRINITY_DN3074_c0_g1_i1_3 | *atpA* | TCTCAGTTAGGTGGAGGGAGTTT | TGCCCGTCGGTAATGGAA |
| TRINITY_DN16239_c0_g1_i3_1 | *psbS* | CTGTTTGTTGGGAGGTTGGC | AGGTTCTATTTCTGTCACTGGGAT |
| TRINITY_DN15612_c0_g1_i2_3 | *LHCA2* | TGCAGGATCGGACCCAGAG | TCATTTCCACCACGAAGAGC |
| TRINITY_DN17085_c0_g1_i3_3 | *LHCB4* | CCGCCAAAGAAGGGTCAA | TCCTGGTCGGGAATAAGTGTC |
| TRINITY_DN17677_c0_g1_i3_3 | *LHCB5* | AAAGAACGGAAGGCTGGCTAT | CGATGACGGTGAGGAGGTTG |
| TRINITY_DN17043_c0_g2_i1_3 | *LHCB1* | CCGCCTCGCTATGTTCTCC | GATGGTCAGCCAGGTTCTCAA |
| TRINITY_DN18301_c0_g1_i2_1 | *CHS* | CTCCGACTGGAACGAACTCTT | GCACGCTCTTGAGAACGACA |
| TRINITY_DN16018_c0_g1_i1_2 | *LAR* | AGGAGCCTGCCCTTACTTTCT | TCAGTGTTCTTGGGTCGTCAA |
| TRINITY_DN15877_c0_g1_i3_3 | *F3H* | TGCCCACAACCTGATATGACC | TCGCCAAGATTGACTACGAAAG |
| TRINITY_DN19521_c0_g1_i5_3 | *FLS* | GATCCGACGTTTGCAGACAG | TAAGAGGAAGGGCATTTAGGC |
| TRINITY_DN15803_c0_g1_i1_2 | *CYP73A* | GGTCTCGAACCCGCAACG | GCAATCTCATTCTCCCAAGCAA |
| TRINITY_DN16889_c0_g1_i1_2 | *DXR* | CCAACATCCTGAGGCTGTCA | AGTTTCTCCACCGGCAAATC |
| TRINITY_DN19523_c0_g1_i6_3 | *ispH*, *lytB* | TCTGTGCTCCTTATACTGGTACTCA | GCTCTGGCTCTGGACAATCA |
| TRINITY_DN5865_c0_g1_i1_1 | *DHDDS* | TTTGTGGCTATCTGAAGTTGGTG | CAGTGCTTGGGTAATTGGAGTT |
| TRINITY_DN12884_c0_g1_i2_3 | *DXS* | TTCCTCTACCTCCCAACTACAAAG | TGGCAACCCGAGTCCCT |
| reference gene | *actin* | GTTGCCATTCAAGCCGTTCT | AACAATTTCACGCTCAGCAGTAG |
|  | *CYP* | TCTCGGGCAGCATTTCACGC | AGCCGAAACTGGCGCCAACA |
|  | *UBC* | CTCGCAGAATCATAAAGGAAACAC | CCATTGGATACTCTTCAGGCAAA |

**Supplementary Table 3**. Content of 106 differentially synthesized metabolites (DSMs).

|  | **Metabolite name** | **WCC** | **SCC** |
| --- | --- | --- | --- |
| 1 | Chlorogenic acid | 1.07443 | 0.00994 |
| 2 | Epicatechin | 0.16662 | 0.00428 |
| 3 | Citric acid | 1.65651 | 0.06407 |
| 4 | Lanosterol | 0.74440 | 0.04033 |
| 5 | Uridine 5'-monophosphate | 0.01355 | 0.00074 |
| 6 | Xylonic acid | 0.37975 | 0.02785 |
| 7 | Beta-tocopherol | 0.00147 | 0.00012 |
| 8 | 6-hydroxyflavone-alpha-d-glucoside | 0.04311 | 0.00357 |
| 9 | Erythronic acid | 0.28739 | 0.03063 |
| 10 | 7-(1-methylethyl)-5-oxo-2-amino-5h-chromeno[2,3-b]pyridine-3-carboxylate | 0.00084 | 0.00009 |
| 11 | N'-(1h-indol-3-ylmethylene)benzohydrazide | 0.21036 | 0.02284 |
| 12 | Malonic acid | 0.00012 | 0.00001 |
| 13 | Maleic acid | 0.00260 | 0.00031 |
| 14 | Saccharopine | 0.01264 | 0.00156 |
| 15 | 3-indoleacetonitrile | 0.00089 | 0.00011 |
| 16 | 3,4-dihydroxy-2,5-furandione | 0.00207 | 0.00028 |
| 17 | Citraconic acid | 0.00320 | 0.00048 |
| 18 | L-2-hydroxyglutaric acid | 0.00645 | 0.00100 |
| 19 | Citramalic acid | 0.00767 | 0.00126 |
| 20 | Malic acid | 0.64791 | 0.10648 |
| 21 | Aconitic acid | 0.00112 | 0.00019 |
| 22 | Erythrono-1,4-lactone | 0.00255 | 0.00042 |
| 23 | Ethyl beta-d-glucopyranoside | 1.15062 | 0.19239 |
| 24 | Arachidonic acid | 0.00238 | 0.00040 |
| 25 | Abietic acid | 0.00841 | 0.00142 |
| 26 | D-fructose-6-phosphate | 0.02271 | 0.00387 |
| 27 | Glucose 6-phosphate | 0.06924 | 0.01185 |
| 28 | N-carbamylglutamate | 0.00050 | 0.00009 |
| 29 | 3-(3-hydroxyphenyl)-3-hydroxypropionic acid | 0.02319 | 0.00423 |
| 30 | 2-desoxy-inosose | 0.02635 | 0.00482 |
| 31 | Digalacturonic acid | 0.62449 | 0.12086 |
| 32 | Glutaric acid | 0.00036 | 0.00007 |
| 33 | Melezitose | 1.12974 | 0.22305 |
| 34 | 4-aminobutyric acid | 0.00317 | 0.00064 |
| 35 | Alpha-tocopherol | 0.37045 | 0.07555 |
| 36 | Beta-gentiobiose | 0.02581 | 0.00560 |
| 37 | 2-oxo-propanoic acid | 0.02387 | 0.00519 |
| 38 | 4-nitrophenol | 0.01126 | 0.00273 |
| 39 | Beta-mannosylglycerate | 0.09315 | 0.02315 |
| 40 | Arabino-hexos-2-ulose, bis(dimethyl acetal) | 0.00410 | 0.00102 |
| 41 | 4-aminophenol | 0.00144 | 0.00036 |
| 42 | Urocanic acid | 0.00038 | 0.00010 |
| 43 | Catechin | 0.00952 | 0.00248 |
| 44 | Succinic acid | 0.00476 | 0.00125 |
| 45 | Tranexamic acid | 0.00087 | 0.00024 |
| 46 | N-acetylglutamate | 0.00070 | 0.00019 |
| 47 | D-fructose-1-phosphate | 0.00016 | 0.00005 |
| 48 | Ascorbic acid | 0.04014 | 0.01140 |
| 49 | Nicotianamine | 0.00098 | 0.00028 |
| 50 | Epsilon-caprolactam | 0.01267 | 0.00362 |
| 51 | Dehydroascorbic acid | 1.12357 | 0.32221 |
| 52 | Isocitric acid | 0.00064 | 0.00018 |
| 53 | 3-pyridinol | 0.11176 | 0.03333 |
| 54 | Methyl beta-d-glucopyranoside | 0.26673 | 0.08621 |
| 55 | Glycerol 3-phosphate | 0.00826 | 0.00268 |
| 56 | D-fructose | 0.00707 | 0.00233 |
| 57 | L-tyrosine | 0.00300 | 0.00099 |
| 58 | Galactonic acid | 0.04985 | 0.01658 |
| 59 | 2,4-dihydroxy-pentanedioic acid | 0.00346 | 0.00118 |
| 60 | Cholic acid | 0.01859 | 0.00664 |
| 61 | Glucose | 2.10407 | 0.75166 |
| 62 | Indole-3-lactate | 0.08230 | 0.03025 |
| 63 | Altrose | 0.07212 | 0.02826 |
| 64 | Squalene | 0.10123 | 0.04002 |
| 65 | Uric acid | 0.00207 | 0.00083 |
| 66 | 1,3-dihydroxypyridine | 0.00187 | 0.00076 |
| 67 | Arabitol | 0.00830 | 0.00338 |
| 68 | Ergosterol | 0.02066 | 0.00846 |
| 69 | 6-o-methyl-alpha-d-glucopyranose | 3.15482 | 1.34377 |
| 70 | 1-monoolein | 0.04580 | 0.01961 |
| 71 | D-myo-inositol 4-phosphate | 0.00100 | 0.00043 |
| 72 | Glycolic acid | 0.00211 | 0.00091 |
| 73 | Maltotriose | 1.53109 | 0.66171 |
| 74 | Methanephosphonothioic acid | 0.09046 | 0.03930 |
| 75 | Glycerol-3-galactoside | 0.11607 | 0.05218 |
| 76 | Oxoproline | 0.09032 | 0.04107 |
| 77 | 5-hydroxy-3-indoleacetic acid | 0.00819 | 0.00374 |
| 78 | Piceatannol | 0.08761 | 0.03999 |
| 79 | Glucose-1-phosphate | 0.41285 | 0.19021 |
| 80 | 2-deoxy-d-erythro-pentitol | 0.00235 | 0.00115 |
| 81 | N-acetyl-5-hydroxytryptamine | 0.30726 | 0.15144 |
| 82 | 2,6-diaminopimelic acid | 0.00045 | 0.00094 |
| 83 | Tartaric acid | 0.00511 | 0.01080 |
| 84 | Homoserine | 0.01401 | 0.02971 |
| 85 | 5-hydroxypipecolic acid | 0.00057 | 0.00121 |
| 86 | Saccharic acid | 0.03851 | 0.08297 |
| 87 | Pyruvic acid | 0.00363 | 0.00786 |
| 88 | Cellobiose | 0.00094 | 0.00205 |
| 89 | Quinic acid | 2.89210 | 7.04726 |
| 90 | L-alanine | 0.01960 | 0.04949 |
| 91 | Serine | 0.01213 | 0.03157 |
| 92 | D-erythro-sphingosine | 0.14247 | 0.37225 |
| 93 | L-isoleucine | 0.00229 | 0.00602 |
| 94 | 1,2,4-butanetriol | 0.00909 | 0.02389 |
| 95 | L-arabitol | 0.00518 | 0.01449 |
| 96 | Aloeemodin | 0.00011 | 0.00031 |
| 97 | Alpha tocopherol | 0.00069 | 0.00209 |
| 98 | Boric acid | 0.00419 | 0.01389 |
| 99 | L-valine | 0.00332 | 0.01178 |
| 100 | Galactinol | 0.03545 | 0.19148 |
| 101 | Adenosine-5'-monophosphate | 0.04249 | 0.24193 |
| 102 | Dodecanoic acid, isopropanol ester | 0.00015 | 0.00088 |
| 103 | Hypoxanthine | 0.00027 | 0.00173 |
| 104 | Glycyl tyrosine | 0.06464 | 0.43826 |
| 105 | Beta-hydroxymyristic acid | 0.00018 | 0.00128 |
| 106 | N-acetylmannosamine | 0.16120 | 8.08462 |

**Supplementary Table 4**. A total of 63 differentially synthesized metabolites (DEMs) were enriched in 69 metabolic pathways.

|  | **Metabolite name** | **Annotation** |
| --- | --- | --- |
| 1 | 2,6-diaminopimelic acid | Lysine biosynthesis |
| 2 | 3-indoleacetonitrile | Tryptophan metabolism |
| 3 | 4-aminobutyric acid | Alanine, aspartate and glutamate metabolism; Butanoate metabolism; Nicotinate and nicotinamide metabolism; Arginine and proline metabolism; beta-alanine metabolism |
| 4 | 5-hydroxy-3-indoleacetic acid | Tryptophan metabolism |
| 5 | Abietic acid | Diterpenoid biosynthesis |
| 6 | Aconitic acid | Citrate cycle (TCA cycle); Glyoxylate and dicarboxylate metabolism; C5-Branched dibasic acid metabolism |
| 7 | Adenosine-5'-monophosphate | Purine metabolism; Zeatin biosynthesis |
| 8 | Alpha-tocopherol | Ubiquinone and other terpenoid-quinone biosynthesis |
| 9 | Arabitol | Pentose and glucuronate interconversions |
| 10 | Arachidonic acid | Linoleic acid metabolism; Biosynthesis of unsaturated fatty acids; Arachidonic acid metabolism |
| 11 | Ascorbic acid | Ascorbate and aldarate metabolism |
| 12 | Beta-tocopherol | Ubiquinone and other terpenoid-quinone biosynthesis |
| 13 | Catechin | Flavonoid biosynthesis |
| 14 | Chlorogenic acid | Stilbenoid, diarylheptanoid and gingerol biosynthesis; Flavonoid biosynthesis; Phenylpropanoid biosynthesis |
| 15 | Citraconic acid | Valine, leucine and isoleucine biosynthesis; C5-Branched dibasic acid metabolism |
| 16 | Citric acid | Citrate cycle (TCA cycle); Glyoxylate and dicarboxylate metabolism; Alanine, aspartate and glutamate metabolism |
| 17 | D-erythro-sphingosine | Sphingolipid metabolism |
| 18 | D-fructose | Amino sugar and nucleotide sugar metabolism |
| 19 | D-fructose-1-phosphate | Phenylalanine, tyrosine and tryptophan biosynthesis; Fructose and mannose metabolism |
| 20 | D-fructose-6-phosphate | Carbon fixation in photosynthetic organisms; Galactose metabolism; Starch and sucrose metabolism |
| 21 | D-myo-inositol 4-phosphate | Inositol phosphate metabolism; Phosphatidylinositol signaling system |
| 22 | Dehydroascorbic acid | Ascorbate and aldarate metabolism; Glutathione metabolism |
| 23 | Digalacturonic acid | Pentose and glucuronate interconversions; ABC transporters |
| 24 | Epicatechin | Flavonoid biosynthesis |
| 25 | Ergosterol | Steroid biosynthesis |
| 26 | Galactinol | Galactose metabolism |
| 27 | Galactonic acid | Galactose metabolism |
| 28 | Glucose | Glycolysis/Gluconeogenesis; Pentose phosphate pathway |
| 29 | Glucose 6-phosphate | Starch and sucrose metabolism; Biosynthesis of various secondary metabolites - part 2; Inositol phosphate metabolism |
| 30 | Glucose-1-phosphate | Pentose and glucuronate interconversions; Galactose metabolism; Glycolysis/Gluconeogenesis; Starch and sucrose metabolism; Glycerolipid metabolism; Amino sugar and nucleotide sugar metabolism |
| 31 | Glutaric acid | Lysine degradation; Fatty acid degradation |
| 32 | Glycerol 3-phosphate | ABC transporters; Glycerolipid metabolism; Glycerophospholipid metabolism |
| 33 | Glycolic acid | Fatty acid degradation |
| 34 | Homoserine | Sulfur metabolism; Lysine biosynthesis; Cysteine and methionine metabolism; Glycine, serine and threonine metabolism |
| 35 | Hypoxanthine | Purine metabolism |
| 36 | Isocitric acid | Citrate cycle (TCA cycle); Glyoxylate and dicarboxylate metabolism |
| 37 | L-2-hydroxyglutaric acid | Lysine degradation |
| 38 | L-alanine | Alanine, aspartate and glutamate metabolism; Carbon fixation in photosynthetic organisms; Aminoacyl-tRNA biosynthesis; Cysteine and methionine metabolism; Taurine and hypotaurine metabolism; ABC transporters; Biosynthesis of various secondary metabolites - part 2; Sulfur relay system; Selenocompound metabolism |
| 39 | L-arabitol | Pentose and glucuronate interconversions |
| 40 | L-isoleucine | Valine, leucine and isoleucine biosynthesis; Aminoacyl-tRNA biosynthesis; Cyanoamino acid metabolism; Glucosinolate biosynthesis; Valine, leucine and isoleucine degradation; Tropane, piperidine and pyridine alkaloid biosynthesis |
| 41 | L-tyrosine | Aminoacyl-tRNA biosynthesis; Cyanoamino acid metabolism; Phenylalanine, tyrosine and tryptophan biosynthesis; Monobactam biosynthesis; Tyrosine metabolism; Phenylalanine metabolism; Thiamine metabolism; Ubiquinone and other terpenoid-quinone biosynthesis; Glucosinolate biosynthesis; Biosynthesis of various secondary metabolites - part 2; Phenylpropanoid biosynthesis; Betalain biosynthesis; Isoquinoline alkaloid biosynthesis |
| 42 | L-valine | Valine, leucine and isoleucine biosynthesis; Aminoacyl-tRNA biosynthesis; Cyanoamino acid metabolism; ABC transporters; Pantothenate and CoA biosynthesis; Glucosinolate biosynthesis; Valine, leucine and isoleucine degradation |
| 43 | Lanosterol | Steroid biosynthesis |
| 44 | Maleic acid | Butanoate metabolism; Nicotinate and nicotinamide metabolism; Tyrosine metabolism |
| 45 | Malic acid | Citrate cycle (TCA cycle); Glyoxylate and dicarboxylate metabolism; Carbon fixation in photosynthetic organisms; Pyruvate metabolism |
| 46 | N-acetyl-5-hydroxytryptamine | Tryptophan metabolism |
| 47 | N-acetylglutamate | Arginine biosynthesis |
| 48 | N-acetylmannosamine | Amino sugar and nucleotide sugar metabolism |
| 49 | N-carbamylglutamate | Histidine metabolism |
| 50 | Oxoproline | Glutathione metabolism |
| 51 | Piceatannol | Stilbenoid, diarylheptanoid and gingerol biosynthesis |
| 52 | Pyruvic acid | Citrate cycle (TCA cycle); Glyoxylate and dicarboxylate metabolism; Alanine, aspartate and glutamate metabolism; Pentose and glucuronate interconversions; Valine, leucine and isoleucine biosynthesis; Carbon fixation in photosynthetic organisms; Butanoate metabolism; Ascorbate and aldarate metabolism; Nicotinate and nicotinamide metabolism; Glycolysis/Gluconeogenesis; Pyruvate metabolism; C5-Branched dibasic acid metabolism; Cysteine and methionine metabolism; Monobactam biosynthesis; Tyrosine metabolism; Glycine, serine and threonine metabolism; Taurine and hypotaurine metabolism; Phenylalanine metabolism; Pantothenate and CoA biosynthesis; Thiamine metabolism; Pentose phosphate pathway; Arginine and proline metabolism; Terpenoid backbone biosynthesis; Phosphonate and phosphinate metabolism |
| 53 | Quinic acid | Phenylalanine, tyrosine and tryptophan biosynthesis |
| 54 | Saccharic acid | Ascorbate and aldarate metabolism |
| 55 | Saccharopine | Lysine degradation; Lysine biosynthesis |
| 56 | Serine | Glyoxylate and dicarboxylate metabolism; Aminoacyl-tRNA biosynthesis; Cyanoamino acid metabolism; Sulfur metabolism; Cysteine and methionine metabolism; Monobactam biosynthesis; Glycine, serine and threonine metabolism; Sphingolipid metabolism |
| 57 | Squalene | Steroid biosynthesis; Sesquiterpenoid and triterpenoid biosynthesis |
| 58 | Succinic acid | Citrate cycle (TCA cycle); Glyoxylate and dicarboxylate metabolism; Alanine, aspartate and glutamate metabolism; Butanoate metabolism; Lysine degradation; Nicotinate and nicotinamide metabolism; Pyruvate metabolism; Sulfur metabolism; Tyrosine metabolism; Phenylalanine metabolism; Oxidative phosphorylation; Propanoate metabolism |
| 59 | Tartaric acid | Glyoxylate and dicarboxylate metabolism |
| 60 | Uric acid | Purine metabolism |
| 61 | Uridine 5'-monophosphate | Pyrimidine metabolism |
| 62 | Urocanic acid | Histidine metabolism |
| 63 | Xylonic acid | Pentose and glucuronate interconversions |

**Supplementary Table 5**. A total of 44 differentially synthesized metabolites (DEMs) were enriched in 45 metabolic pathways.

|  | **Metabolites** | **Annotation** |
| --- | --- | --- |
| 1 | (-)-cis-Carveol | Limonene and pinene degradation |
| 2 | (S)-2-Acetolactate | Valine, leucine and isoleucine biosynthesis; Pantothenate and CoA biosynthesis; C5-Branched dibasic acid metabolism; Butanoate metabolism |
| 3 | 2-Isopropyl-3-oxosuccinate | Valine, leucine and isoleucine biosynthesis |
| 4 | 2-Phospho-D-glyceric acid | Pentose phosphate pathway; Glyoxylate and dicarboxylate metabolism; Glycolysis/Gluconeogenesis; Glycerolipid metabolism; Glycine, serine and threonine metabolism |
| 5 | 3-Hydroxybenzoic acid | Phenylalanine, tyrosine and tryptophan biosynthesis |
| 6 | 3,4-Dihydroxyphenylglycol | Tyrosine metabolism |
| 7 | 4-Hydroxybenzoic acid | Ubiquinone and other terpenoid-quinone biosynthesis; Folate biosynthesis |
| 8 | 4-Hydroxycinnamic acid | Tyrosine metabolism; Phenylpropanoid biosynthesis; Ubiquinone and other terpenoid-quinone biosynthesis; Biosynthesis of various secondary metabolites - part 2; Isoquinoline alkaloid biosynthesis |
| 9 | 5-Aminoimidazole ribonucleotide | Thiamine metabolism; Purine metabolism |
| 10 | 6-Phosphogluconic acid | Pentose phosphate pathway |
| 11 | Adenine | Purine metabolism; Zeatin biosynthesis |
| 12 | All-trans-heptaprenyl diphosphate | Terpenoid backbone biosynthesis |
| 13 | alpha-Humulene | Sesquiterpenoid and triterpenoid biosynthesis |
| 14 | Alpha-Linolenic acid | alpha-Linolenic acid metabolism; Biosynthesis of unsaturated fatty acids |
| 15 | Ascorbic acid | Ascorbate and aldarate metabolism |
| 16 | Catechin | Flavonoid biosynthesis |
| 17 | Chalconaringenin | Flavonoid biosynthesis |
| 18 | Chlorogenic acid | Flavonoid biosynthesis; Stilbenoid, diarylheptanoid and gingerol biosynthesis; Phenylpropanoid biosynthesis |
| 19 | Cosmosiin | Flavone and flavonol biosynthesis |
| 20 | Cyanidin 3-glucoside | Anthocyanin biosynthesis |
| 21 | D-Sedoheptulose 7-phosphate | Pentose phosphate pathway; Carbon fixation in photosynthetic organisms |
| 22 | Dattelic acid | Flavonoid biosynthesis; Stilbenoid, diarylheptanoid and gingerol biosynthesis; Phenylpropanoid biosynthesis |
| 23 | Dehydroascorbic acid | Ascorbate and aldarate metabolism; Glutathione metabolism |
| 24 | Flavin Mononucleotide | Oxidative phosphorylation; Riboflavin metabolism |
| 25 | Galactaric acid | Ascorbate and aldarate metabolism |
| 26 | Glutaric acid | Fatty acid degradation; Lysine degradation |
| 27 | Isocitric acid | Glyoxylate and dicarboxylate metabolism; Citrate cycle (TCA cycle) |
| 28 | Isowertin 2''-rhamnoside | Flavone and flavonol biosynthesis |
| 29 | L-Valine | Valine, leucine and isoleucine biosynthesis; Pantothenate and CoA biosynthesis; Cyanoamino acid metabolism; Valine, leucine and isoleucine degradation; Aminoacyl-tRNA biosynthesis; Glucosinolate biosynthesis; ABC transporters |
| 30 | Leucopelargonidin | Flavonoid biosynthesis |
| 31 | Linamarin | Cyanoamino acid metabolism |
| 32 | Mevalonic acid-5P | Terpenoid backbone biosynthesis |
| 33 | N-Acetylornithine | Arginine biosynthesis |
| 34 | N-Jasmonoylisoleucine | Plant hormone signal transduction |
| 35 | Naringenin | Flavonoid biosynthesis |
| 36 | Naringin | Flavonoid biosynthesis |
| 37 | Norepinephrine | Tyrosine metabolism |
| 38 | p-Hydroxyphenylacetic acid | Tyrosine metabolism; Phenylalanine metabolism |
| 39 | Perillic acid | Limonene and pinene degradation |
| 40 | Phenylacetic acid | Phenylalanine metabolism |
| 41 | Phlorizin | Flavonoid biosynthesis |
| 42 | Quercetin | Flavonoid biosynthesis; Flavone and flavonol biosynthesis |
| 43 | Salicylic acid | Plant hormone signal transduction; Phenylalanine metabolism |
| 44 | Trehalose 6-phosphate | Starch and sucrose metabolism |

**Supplementary Table 8.** Statistics of splicing results.

| **Term** | **All** | **≥ 500 bp** | **≥ 1000 bp** | **N50 (bp)** | **Total length (bp)** | **Average length (bp)** |
| --- | --- | --- | --- | --- | --- | --- |
| Unigene | 33,063 | 22,881 | 15,310 | 1965 | 42,468,815 | 1284.48 |

**Supplementary Table 9.** Annotation statistics table for each database.

| **Annotated database** | **Annotated number** | **300 bp ≤ length < 1000 bp** | **Length ≥ 1000 bp** |
| --- | --- | --- | --- |
| NR | 21,309 (64.45%) | 7092 (21.45%) | 14,217 (43.00%) |
| SwissProt | 16,470 (49.81%) | 4800 (14.52%) | 11,670 (35.30%) |
| KEGG | 4640 (14.03%) | 1249 (3.78%) | 3391 (10.26%) |
| KOG | 12,183 (36.85%) | 3608 (10.91%) | 8575 (25.94%) |
| eggNOG | 19,833 (59.99%) | 6113 (18.49%) | 13,720 (41.50%) |
| GO | 13,654 (41.30%) | 3574 (10.81%) | 10,080 (30.49%) |
| Pfam | 16,691 (50.48%) | 4315 (13.05%) | 12,376 (37.43%) |

**Supplementary Table 10.** KEGG analysis of the top 20 metabolites and unigenes.

|  | **Metabolites/unigenes** | **Pathway** | **Pathway definition** |
| --- | --- | --- | --- |
| metabolites | Cosmosiin | ath00944 | Flavone and flavonol biosynthesis |
|  | Quercetin | ath00941 | Flavonoid biosynthesis |
|  | Catechin | ath00941 | Flavonoid biosynthesis |
|  | Naringin | ath00941 | Flavonoid biosynthesis |
|  | Chlorogenic acid | ath00941; ath00945; ath00940 | Flavonoid biosynthesis; Stilbenoid, diarylheptanoid and gingerol biosynthesis; Phenylpropanoid biosynthesis |
|  | D-Sedoheptulose 7-phosphate | ath00030; ath00710 | Pentose phosphate pathway; Carbon fixation in photosynthetic organisms |
|  | Trehalose 6-phosphate | ath00500 | Starch and sucrose metabolism |
|  | 4-Hydroxycinnamic acid | ath00350; ath00940; ath00130; ath00998; ath00950 | Tyrosine metabolism; Phenylpropanoid biosynthesis; Ubiquinone and other terpenoid-quinone biosynthesis; Biosynthesis of various secondary metabolites - part 2; Isoquinoline alkaloid biosynthesis |
|  | Mevalonic acid-5P | ath00900 | Terpenoid backbone biosynthesis |
|  | Adenine | ath00230; ath00908 | Purine metabolism; Zeatin biosynthesis |
|  | alpha-Humulene | ath00909 | Sesquiterpenoid and triterpenoid biosynthesis |
|  | 2-Isopropyl-3-oxosuccinate | ath00290 | Valine, leucine and isoleucine biosynthesis |
|  | (S)-2-Acetolactate | ath00290; ath00770; ath00660; ath00650 | Valine, leucine and isoleucine biosynthesis; Pantothenate and CoA biosynthesis; C5-Branched dibasic acid metabolism; Butanoate metabolism |
|  | 3,4-Dihydroxyphenylglycol | ath00350 | Tyrosine metabolism |
|  | p-Hydroxyphenylacetic acid | ath00350; ath00360 | Tyrosine metabolism; Phenylalanine metabolism |
|  | Glutaric acid | ath00310; ath00071 | Lysine degradation; Fatty acid degradation |
|  | N-Acetylornithine | ath00220 | Arginine biosynthesis |
|  | Isocitric acid | ath00020; ath00630 | Citrate cycle (TCA cycle); Glyoxylate and dicarboxylate metabolism |
|  | Alpha-Linolenic acid | ath00592; ath01040 | alpha-Linolenic acid metabolism; Biosynthesis of unsaturated fatty acids |
|  | Ascorbic acid | ath00053 | Ascorbate and aldarate metabolism |
| unigenes | *TRINITY_DN9207_c0_g1_i1_2* | ko00195 | Photosynthesis |
|  | *TRINITY_DN12449_c0_g1_i1_1* |  |  |
|  | *TRINITY_DN799_c0_g1_i1_3* |  |  |
|  | *TRINITY_DN14460_c0_g1_i1_3* |  |  |
|  | *TRINITY_DN16127_c0_g1_i1_2* |  |  |
|  | *TRINITY_DN8226_c0_g1_i2_2* |  |  |
|  | *TRINITY_DN17864_c0_g2_i2_2* |  |  |
|  | *TRINITY_DN22031_c0_g1_i1_1* |  |  |
|  | *TRINITY_DN11939_c0_g2_i2_1* |  |  |
|  | *TRINITY_DN8492_c0_g1_i1_1* |  |  |
|  | *TRINITY_DN17043_c0_g2_i1_3* | ko00196 | Photosynthesis-antenna proteins |
|  | *TRINITY_DN16105_c0_g1_i1_1* |  |  |
|  | *TRINITY_DN10469_c0_g1_i3_2* |  |  |
|  | *TRINITY_DN16406_c0_g1_i2_3* |  |  |
|  | *TRINITY_DN15800_c0_g1_i2_2* | ko00380; ko00630; ko04016; ko04146 | Tryptophan metabolism; Glyoxylate and dicarboxylate metabolism; MAPK signaling pathway - plant; Peroxisome |
|  | *TRINITY_DN12955_c0_g2_i2_3* | ko00630; ko00710 | Arginine biosynthesis; Alanine, aspartate and glutamate metabolism; Glycine, serine and threonine metabolism; Glyoxylate and dicarboxylate metabolism; Carbon fixation in photosynthetic organisms |
|  | *TRINITY_DN12955_c0_g1_i4_3* |  |  |
|  | *TRINITY_DN18301_c0_g1_i2_1* | ko00941; ko04712 | Flavonoid biosynthesis; Circadian rhythm-plant |
|  | *TRINITY_DN14673_c0_g1_i1_2* | ko00010; ko00030; ko00051; ko00710 | Glycolysis/Gluconeogenesis; Pentose phosphate pathway; Fructose and mannose metabolism; Carbon fixation in photosynthetic organisms |
|  | *TRINITY_DN16296_c0_g1_i5_1* | ko03013 | RNA transport |

**Supplementary Table 11.** Unigenes involved in photosynthesis and photosynthesis-antenna proteins.

| Gene | **Unigenes** | SCC | WCC |
| --- | --- | --- | --- |
| Photosynthesis | | | |
| *ATPF0B, atpF* | *TRINITY_DN9390_c0_g1_i1_2* | 1031.113 | 544.511 |
| *ATPF1A, atpA* | *TRINITY_DN3074_c0_g1_i1_3* | 226.783 | 40.623 |
| *ATPF1B, atpD* | *TRINITY_DN23882_c0_g1_i1_3* | 5.227 | 1.816 |
| *ATPF1D, atpH* | *TRINITY_DN14390_c0_g2_i1_3* | 472.872 | 163.790 |
| *ATPF1E, atpC* | *TRINITY_DN22374_c0_g1_i1_2* | 2.939 | 0.237 |
| *ATPF1G, atpG* | *TRINITY_DN21365_c0_g1_i1_3* | 566.230 | 229.262 |
| *petE* | *TRINITY_DN799_c0_g1_i1_3* | 4350.303 | 257.911 |
| *petF* | *TRINITY_DN12386_c0_g2_i1_2* | 587.886 | 181.454 |
|  | *TRINITY_DN15346_c0_g1_i1_1* | 68.613 | 13.802 |
|  | *TRINITY_DN16127_c0_g1_i1_2* | 2994.524 | 780.754 |
| *petH* | *TRINITY_DN15935_c0_g1_i2_1* | 815.824 | 281.625 |
|  | *TRINITY_DN7760_c0_g1_i1_1* | 23.564 | 59.481 |
| *petJ* | *TRINITY_DN15629_c0_g1_i2_1* | 47.537 | 16.997 |
| *psaD* | *TRINITY_DN22031_c0_g1_i1_1* | 2753.416 | 210.313 |
| *psaE* | *TRINITY_DN14460_c0_g1_i1_3* | 4053.605 | 456.665 |
| *psaF* | *TRINITY_DN5246_c0_g1_i1_2* | 1627.697 | 266.014 |
| *psaG* | *TRINITY_DN10442_c0_g1_i1_2* | 1934.192 | 163.764 |
| *psaH* | *TRINITY_DN11939_c0_g2_i2_1* | 2252.033 | 357.942 |
|  | *TRINITY_DN7713_c0_g1_i2_2* | 394.001 | 39.038 |
| *psaK* | *TRINITY_DN8043_c0_g1_i2_3* | 779.059 | 36.937 |
| *psaL* | *TRINITY_DN8226_c0_g1_i2_2* | 3066.087 | 704.905 |
| *psaN* | *TRINITY_DN11807_c0_g1_i1_2* | 2291.134 | 145.148 |
| *psaO* | *TRINITY_DN8492_c0_g1_i1_1* | 2508.696 | 23.207 |
| *psb27* | *TRINITY_DN7646_c0_g1_i2_1* | 324.446 | 3.886 |
| *psb28* | *TRINITY_DN17552_c0_g1_i1_3* | 132.633 | 56.263 |
| *psbA* | *TRINITY_DN6890_c0_g1_i1_3* | 23.765 | 120.368 |
| *psbB* | *TRINITY_DN10352_c0_g1_i2_2* | 16.237 | 7.759 |
| *psbI* | *TRINITY_DN21672_c0_g1_i1_1* | 7.701 | 3.193 |
| *psbO* | *TRINITY_DN17864_c0_g2_i2_2* | 2454.532 | 589.179 |
| *psbP* | *TRINITY_DN12449_c0_g1_i1_1* | 4320.260 | 658.362 |
|  | *TRINITY_DN13008_c0_g1_i2_1* | 76.372 | 18.544 |
|  | *TRINITY_DN14524_c0_g1_i3_2* | 24.188 | 6.811 |
| *psbQ* | *TRINITY_DN16711_c0_g2_i2_1* | 1926.207 | 241.955 |
|  | *TRINITY_DN16711_c0_g3_i1_1* | 57.626 | 1.946 |
| *psbR* | *TRINITY_DN9207_c0_g1_i1_2* | 4279.616 | 1462.793 |
| *psbS* | *TRINITY_DN16239_c0_g1_i3_1* | 438.103 | 1138.172 |
| *psbW* | *TRINITY_DN18484_c0_g1_i1_2* | 1289.400 | 353.186 |
| *psbY* | *TRINITY_DN14870_c0_g2_i1_2* | 1544.444 | 373.882 |
| *psbZ* | *TRINITY_DN1935_c0_g1_i1_2* | 18.578 | 7.901 |
| Photosynthesis-antenna proteins | | | |
| *LHCA1* | *TRINITY_DN9810_c0_g1_i4_1* | 334.399 | 38.705 |
| *LHCA2* | *TRINITY_DN15612_c0_g1_i2_3* | 1307.216 | 128.767 |
| *LHCA3* | *TRINITY_DN5326_c0_g1_i1_1* | 1395.184 | 84.064 |
| *LHCA4* | *TRINITY_DN16406_c0_g1_i2_3* | 3047.307 | 69.822 |
|  | *TRINITY_DN16406_c0_g2_i1_3* | 11.878 | 0.169 |
| *LHCB1* | *TRINITY_DN16105_c0_g1_i1_1* | 5570.274 | 50.228 |
|  | *TRINITY_DN17043_c0_g2_i1_3* | 8163.685 | 1169.154 |
| *LHCB2* | *TRINITY_DN10469_c0_g1_i3_2* | 4092.938 | 17.703 |
| *LHCB3* | *TRINITY_DN18493_c0_g1_i9_1* | 73.205 | 1.231 |
|  | *TRINITY_DN5493_c0_g1_i1_2* | 1859.453 | 4.443 |
| *LHCB4* | *TRINITY_DN17085_c0_g1_i3_3* | 866.955 | 256.830 |
|  | *TRINITY_DN23197_c0_g1_i1_3* | 2000.834 | 32.693 |
| *LHCB5* | *TRINITY_DN15286_c0_g1_i4_2* | 2197.964 | 249.624 |
|  | *TRINITY_DN16314_c0_g1_i1_1* | 8.618 | 0.038 |
|  | *TRINITY_DN17677_c0_g1_i3_3* | 747.276 | 104.794 |
| *LHCB6* | *TRINITY_DN15215_c0_g1_i1_2* | 1928.090 | 49.989 |
| *LHCB7* | *TRINITY_DN14428_c0_g1_i1_3* | 18.391 | 8.115 |

**Supplementary Table 12.** Metabolites involved in the terpenoid backbone biosynthesis pathway.

| **Metabolites** | **SCC** | WCC |
| --- | --- | --- |
| all-trans-heptaprenyl diphosphate | 123398.951 | 24414.524 |
| mevalonic acid-5P | 541134.525 | 136929.761 |
| pyruvic acid | 0.008 | 0.004 |

**Supplementary Table 13.** Unigenes involved in the terpenoid backbone biosynthesis pathway.

| KEGG gene name | Unigenes | SCC | WCC |
| --- | --- | --- | --- |
| *DHDDS, RER2, SRT1* | *TRINITY_DN13237_c0_g1_i2_3* | 2184.947 | 7.445 |
|  | *TRINITY_DN17497_c0_g1_i3_3* | 2.394 | 20.943 |
|  | *TRINITY_DN25256_c0_g1_i1_1* | 0.938 | 0.089 |
|  | *TRINITY_DN5865_c0_g1_i1_1* | 502.807 | 44.138 |
| *dxr* | *TRINITY_DN16889_c0_g1_i1_2* | 77.590 | 40.064 |
| *dxs* | *TRINITY_DN11076_c0_g1_i1_2* | 5.619 | 23.147 |
|  | *TRINITY_DN12884_c0_g1_i2_3* | 58.587 | 19.663 |
|  | *TRINITY_DN17740_c0_g1_i5_1* | 7.500 | 28.364 |
| *atoB* | *TRINITY_DN17193_c0_g1_i1_1* | 2.143 | 17.595 |
|  | *TRINITY_DN17193_c0_g2_i3_1* | 78.532 | 187.982 |
| *HMGCS* | *TRINITY_DN18275_c0_g1_i3_2* | 30.344 | 102.358 |
|  | *TRINITY_DN23213_c0_g1_i1_2* | 1.350 | 0.000 |
| *FNTA* | *TRINITY_DN16330_c0_g1_i7_2* | 36.332 | 17.174 |
| *GGPS* | *TRINITY_DN13187_c0_g1_i1_3* | 0.127 | 2.943 |
|  | *TRINITY_DN18695_c0_g1_i1_3* | 22.130 | 10.767 |
|  | *TRINITY_DN2362_c0_g1_i1_3* | 2.183 | 0.115 |
|  | *TRINITY_DN6505_c0_g1_i1_1* | 0.000 | 2.209 |
| *HMGCR* | *TRINITY_DN6502_c0_g1_i1_3* | 3.394 | 0.544 |
| *ispF* | *TRINITY_DN14639_c0_g1_i1_1* | 20.775 | 62.991 |
| *ispH, lytB* | *TRINITY_DN18491_c0_g1_i5_2* | 40.411 | 4.869 |
|  | *TRINITY_DN19176_c0_g1_i8_1* | 60.341 | 6.609 |
|  | *TRINITY_DN19523_c0_g1_i6_3* | 420.048 | 33.314 |
| *ispS* | *TRINITY_DN15589_c0_g1_i1_1* | 26.184 | 1.653 |
|  | *TRINITY_DN16618_c0_g1_i5_1* | 0.114 | 6.240 |
|  | *TRINITY_DN18722_c0_g1_i4_2* | 1.020 | 16.940 |
|  | *TRINITY_DN19835_c0_g1_i9_3* | 0.492 | 29.453 |
| *MVD, mvaD* | *TRINITY_DN20385_c0_g1_i1_1* | 32.378 | 71.772 |
| *STE24* | *TRINITY_DN3282_c0_g1_i1_2* | 52.900 | 122.689 |

**Supplementary Table 14.** Unigenes involved in terpenoid-related biosynthesis pathways.

| Pathway | KEGG gene name | Unigenes | SCC | WCC |
| --- | --- | --- | --- | --- |
| monoterpenoid biosynthesis | *alpha-terpineol synthase (ATESY)* | *TRINITY_DN18306_c0_g1_i15_1* | 7.951 | 0.126 |
|  | *(-)-alpha-terpineol synthase* | *TRINITY_DN18468_c0_g1_i1_3* | 111.514 | 0.903 |
|  | *MD* | *TRINITY_DN19154_c0_g1_i16_2* | 1.987 | 7.591 |
|  |  | *TRINITY_DN19933_c0_g1_i7_3* | 21.548 | 72.914 |
|  |  | *TRINITY_DN6183_c0_g1_i1_2* | 3.915 | 21.577 |
|  |  | *TRINITY_DN9942_c0_g1_i2_3* | 0.371 | 3.256 |
| diterpenoid biosynthesis | *GA20ox* | *TRINITY_DN21646_c0_g1_i1_2* | 0.181 | 2.956 |
|  | *(13E)-labda-7,13-dien-15-ol synthase* | *TRINITY_DN15238_c0_g1_i9_3* | 4.477 | 0.456 |
|  |  | *TRINITY_DN15767_c0_g1_i3_1* | 32.191 | 0.882 |
|  |  | *TRINITY_DN17858_c0_g1_i16_2* | 14.707 | 0.973 |
|  |  | *TRINITY_DN18051_c0_g1_i12_1* | 27.083 | 1.857 |
|  | *GA3, CYP701* | *TRINITY_DN16316_c0_g1_i1_2* | 7.112 | 0.009 |
|  |  | *TRINITY_DN18229_c0_g1_i1_1* | 15.823 | 3.200 |
|  |  | *TRINITY_DN19022_c0_g1_i10_2* | 25.388 | 0.010 |
| sesquiterpenoid and triterpenoid biosynthesis | *AFS1* | *TRINITY_DN19227_c0_g3_i1_2* | 0 | 9.191 |
|  | *NES1* | *TRINITY_DN18667_c0_g3_i1_2* | 29.437 | 4.295 |
|  | *SQLE, ERG1* | *TRINITY_DN18075_c0_g1_i2_3* | 26.603 | 11.881 |
| carotenoid biosynthesis | *AAO3* | *TRINITY_DN19890_c0_g3_i1_1* | 25.808 | 9.739 |
|  |  | *TRINITY_DN19976_c1_g1_i1_3* | 2.82 | 0.862 |
|  | *CCD8* | *TRINITY_DN17200_c0_g1_i2_1* | 7.543 | 0 |
|  | *crtB* | *TRINITY_DN14040_c0_g1_i2_3* | 51.319 | 174.082 |
|  |  | *TRINITY_DN14820_c0_g1_i1_3* | 21.524 | 4.737 |
|  | *crtZ* | *TRINITY_DN15611_c0_g1_i4_2* | 41.422 | 153.633 |
|  |  | *TRINITY_DN17220_c0_g1_i1_3* | 7.704 | 28.101 |
|  |  | *TRINITY_DN17831_c0_g1_i4_2* | 1.428 | 109.404 |
|  |  | *TRINITY_DN18153_c0_g2_i2_3* | 3.365 | 162.18 |
|  | *CYP707A* | *TRINITY_DN13766_c0_g2_i1_2* | 6.415 | 15.9 |
|  |  | *TRINITY_DN15504_c0_g1_i3_3* | 22.026 | 54.016 |
|  |  | *TRINITY_DN16355_c0_g1_i5_1* | 15.22 | 45.223 |
|  |  | *TRINITY_DN16355_c0_g2_i1_1* | 0.87 | 17.149 |
|  |  | *TRINITY_DN25042_c0_g1_i1_3* | 5.284 | 0 |
|  |  | *TRINITY_DN4618_c0_g1_i1_3* | 1.588 | 0.713 |
|  | *lcyB, crtL1, crtY* | *TRINITY_DN8094_c0_g1_i1_2* | 20.78 | 81.521 |
|  | *lcyE, crtL2* | *TRINITY_DN7488_c0_g1_i2_2* | 73.988 | 37.086 |
|  | *NCED* | *TRINITY_DN16521_c0_g1_i1_2* | 32.141 | 1.286 |
|  |  | *TRINITY_DN18108_c0_g2_i1_1* | 55.757 | 27.537 |
|  |  | *TRINITY_DN18937_c0_g2_i5_2* | 161.981 | 2.133 |
|  |  | *TRINITY_DN20294_c0_g1_i1_1* | 2.366 | 0 |
|  |  | *TRINITY_DN3888_c0_g1_i1_2* | 76.715 | 0.012 |
|  |  | *TRINITY_DN4541_c0_g1_i1_1* | 1.978 | 0 |
|  | *VDE, NPQ1* | *TRINITY_DN6676_c0_g1_i2_1* | 0.035 | 159.495 |
|  | *ZDS, crtQ* | *TRINITY_DN14024_c0_g1_i2_1* | 34.864 | 81.414 |
| brassinosteroid biosynthesis | *CYP734A1, BAS1* | *TRINITY_DN13845_c0_g1_i1_1* | 11.928 | 2.022 |
|  |  | *TRINITY_DN15850_c0_g1_i1_3* | 18.833 | 7.064 |
|  |  | *TRINITY_DN19056_c0_g2_i5_1* | 7.720 | 3.747 |
|  |  | *TRINITY_DN6505_c0_g1_i2_3* | 0.534 | 19.529 |
|  | *CYP90A1, CPD* | *TRINITY_DN8147_c0_g1_i3_3* | 47.465 | 12.308 |
|  | *CYP90B1, DWF4* | *TRINITY_DN15120_c0_g2_i3_3* | 0.352 | 1.291 |
|  |  | *TRINITY_DN16341_c0_g1_i1_1* | 1.493 | 7.406 |
|  |  | *TRINITY_DN16341_c0_g2_i3_1* | 2.656 | 1.327 |
|  |  | *TRINITY_DN4106_c0_g1_i2_1* | 10.433 | 0.916 |
|  |  | *TRINITY_DN6846_c0_g1_i2_2* | 1.306 | 4.089 |
|  | *CYP90D1* | *TRINITY_DN12640_c0_g1_i5_1* | 248.184 | 47.920 |
|  | *CYP92A6* | *TRINITY_DN17801_c1_g4_i1_2* | 3.932 | 0.454 |
|  |  | *TRINITY_DN18661_c0_g5_i1_3* | 33.123 | 0.000 |
|  |  | *TRINITY_DN19154_c0_g2_i1_1* | 215.942 | 30.929 |
|  | *DET2* | *TRINITY_DN25020_c0_g1_i1_3* | 3.303 | 14.285 |
| zeatin biosynthesis | *CISZOG* | *TRINITY_DN16426_c0_g1_i1_1* | 8.871 | 1.623 |
|  |  | *TRINITY_DN7980_c0_g1_i1_2* | 3.643 | 0.467 |
|  | *CKX* | *TRINITY_DN1993_c0_g1_i1_1* | 5.819 | 0.166 |
|  |  | *TRINITY_DN3131_c0_g1_i1_2* | 0.189 | 1.462 |
|  |  | *TRINITY_DN3184_c0_g1_i1_3* | 0.249 | 1.629 |
|  |  | *TRINITY_DN7740_c0_g1_i2_1* | 7.795 | 0.516 |
|  | *CYP735A* | *TRINITY_DN24054_c0_g1_i1_3* | 8.321 | 3.230 |
|  |  | *TRINITY_DN3916_c0_g1_i1_1* | 0.319 | 2.275 |
|  |  | *TRINITY_DN5198_c0_g1_i1_1* | 1.871 | 0.400 |
|  |  | *TRINITY_DN551_c0_g1_i1_2* | 0 | 2.037 |
|  |  | *TRINITY_DN9366_c0_g1_i1_3* | 0.383 | 3.117 |
|  | *UGT73C* | *TRINITY_DN16993_c0_g3_i1_1* | 4.917 | 55.303 |
|  |  | *TRINITY_DN17922_c0_g1_i1_2* | 14.314 | 34.131 |
|  |  | *TRINITY_DN19157_c0_g1_i1_1* | 0.824 | 525.081 |
|  |  | *TRINITY_DN1965_c0_g1_i1_3* | 1.518 | 0.009 |

**Supplementary Table 15.** Unigenes involved in the flavonoid biosynthesis pathway.

| KEGG gene name | Unigenes | SCC | WCC |
| --- | --- | --- | --- |
| *ANR* | *TRINITY_DN14656_c0_g1_i3_3* | 38.809 | 644.013 |
| *ANS* | *TRINITY_DN19073_c0_g1_i4_2* | 123.094 | 952.849 |
| *CHS* | *TRINITY_DN18133_c0_g1_i1_2* | 8.512 | 603.623 |
|  | *TRINITY_DN18133_c0_g2_i2_2* | 0.718 | 306.764 |
|  | *TRINITY_DN18301_c0_g1_i2_1* | 409.749 | 2486.749 |
|  | *TRINITY_DN18429_c0_g2_i3_2* | 444.762 | 1265.968 |
|  | *TRINITY_DN18429_c0_g3_i2_2* | 0.439 | 560.469 |
|  | *TRINITY_DN19332_c0_g1_i9_1* | 88.902 | 1346.446 |
|  | *TRINITY_DN19353_c0_g1_i4_2* | 209.758 | 1575.461 |
|  | *TRINITY_DN19353_c0_g4_i3_2* | 205.119 | 1915.695 |
|  | *TRINITY_DN20047_c0_g1_i7_3* | 7.652 | 264.621 |
|  | *TRINITY_DN20047_c0_g2_i1_3* | 0.305 | 293.026 |
| *CYP73A* | *TRINITY_DN15388_c0_g1_i2_3* | 33.710 | 79.271 |
|  | *TRINITY_DN15421_c0_g1_i1_2* | 43.941 | 252.638 |
|  | *TRINITY_DN15803_c0_g1_i1_2* | 14.474 | 49.289 |
| *CYP75A, F3’5’H* | *TRINITY_DN9564_c0_g1_i1_3* | 0 | 18.009 |
| *CYP75B1, F3’H* | *TRINITY_DN11616_c0_g1_i1_1* | 0.032 | 10.534 |
|  | *TRINITY_DN9520_c0_g1_i5_2* | 9.958 | 341.696 |
| *DFR* | *TRINITY_DN15839_c0_g1_i1_1* | 28.444 | 248.008 |
|  | *TRINITY_DN16628_c0_g1_i6_3* | 45.276 | 295.981 |
|  | *TRINITY_DN19415_c0_g3_i12_1* | 151.014 | 34.664 |
|  | *TRINITY_DN19489_c0_g1_i4_3* | 9.059 | 34.217 |
|  | *TRINITY_DN3460_c0_g1_i1_3* | 8.389 | 1.084 |
| *CCoAOMT* | *TRINITY_DN17771_c1_g1_i5_1* | 22.090 | 66.543 |
|  | *TRINITY_DN19187_c0_g1_i6_3* | 21.537 | 92.262 |
| *HCT* | *TRINITY_DN17171_c0_g1_i1_2* | 36.322 | 9.124 |
|  | *TRINITY_DN17420_c0_g1_i2_1* | 1.092 | 27.133 |
|  | *TRINITY_DN18760_c0_g1_i1_2* | 28.997 | 15.386 |
|  | *TRINITY_DN18883_c0_g1_i1_3* | 10.010 | 21.693 |
|  | *TRINITY_DN22474_c0_g1_i1_1* | 3.931 | 0.509 |
|  | *TRINITY_DN4070_c0_g1_i1_1* | 4.965 | 0.627 |
| *CHI* | *TRINITY_DN8116_c0_g1_i3_2* | 22.574 | 621.339 |
| *F3H* | *TRINITY_DN11984_c0_g1_i1_3* | 52.327 | 0.235 |
|  | *TRINITY_DN12628_c0_g1_i3_1* | 0.040 | 8.397 |
|  | *TRINITY_DN15692_c0_g1_i1_2* | 40.631 | 867.910 |
|  | *TRINITY_DN15877_c0_g1_i3_3* | 18.261 | 634.828 |
|  | *TRINITY_DN19502_c0_g1_i4_1* | 6.755 | 26.845 |
|  | *TRINITY_DN9436_c0_g1_i4_2* | 5.013 | 358.075 |
| *FLS* | *TRINITY_DN12657_c0_g1_i2_2* | 1.000 | 28.797 |
|  | *TRINITY_DN14900_c0_g2_i1_1* | 1.350 | 0.029 |
|  | *TRINITY_DN19521_c0_g1_i5_3* | 2.224 | 131.349 |
|  | *TRINITY_DN19521_c0_g2_i1_3* | 8.778 | 20.767 |
|  | *TRINITY_DN22439_c0_g1_i1_3* | 1.690 | 0 |
|  | *TRINITY_DN23896_c0_g1_i1_1* | 0.155 | 6.048 |
| *LAR* | *TRINITY_DN14021_c0_g1_i2_1* | 9.847 | 2.779 |
|  | *TRINITY_DN16018_c0_g1_i1_2* | 5.645 | 153.907 |
|  | *TRINITY_DN16972_c0_g1_i3_1* | 1.049 | 46.195 |

**Supplementary Table 16.** Metabolites involved in the flavonoid biosynthesis pathway.

| **Metabolites** | **SCC** | WCC |
| --- | --- | --- |
| Catechin_1 | 0.002 | 0.010 |
| Chlorogenic acid_1 | 0.010 | 1.074 |
| Epicatechin | 0.004 | 0.167 |
| Catechin | 21,264.574 | 3,486,882.198 |
| Chalconaringenin | 9780.522 | 409,434.820 |
| Caffeoyl quinic acid | 113,323.311 | 5,377,368.291 |
| Caffeoyl shikimic acid | 13,966.631 | 216,192.816 |
| Leucopelargonidin | 3081.811 | 486,292.679 |
| Naringenin | 5.1875E-05 | 317,852.896 |
| Naringin | 8122.891 | 546,805.234 |
| Phlorizin | 61,035.043 | 345,462.339 |
| Quercetin | 420,777.808 | 4,088,082.198 |
